# Professional Independence as the Foundation of Modern Pharmacy Practice: A Comparative Analysis of Deontological Frameworks Across 30 European Jurisdictions

**DOI:** 10.3390/healthcare14183028

**Published:** 2026-09-15

**Authors:** Hajnal Finta, Marius Călin Chereches, Ioana-Maria Stroia, Andrei Șuteu-Albu, Sonia Bianca Blaj, Daniela-Lucia Muntean

**Affiliations:** 1 Department of Public Health and Health Management, George Emil Palade University of Medicine, Pharmacy, Science and Technology of Targu Mures, Gheorghe Marinescu Street No. 38, 540139 Targu Mures, Romania; hajnal.finta@umfst.ro; 2 Faculty of Pharmacy, George Emil Palade University of Medicine, Pharmacy, Science and Technology of Targu Mures, 540139 Targu Mures, Romania; marius.chereches@umfst.ro (M.C.C.); daniela.muntean@umfst.ro (D.-L.M.); 3 Department of Law and Public Administration, George Emil Palade University of Medicine, Pharmacy, Science and Technology of Targu Mures, Gheorghe Marinescu Street No. 38, 540139 Targu Mures, Romania; sonia.blaj@umfst.ro

**Keywords:** pharmaceutical care, professional independence, deontological code, pharmacist, Europe, digital health, patient-centered care, clinical pharmacist, comparative analysis, public health

## Abstract

**Highlights:**

**What are the main findings?**

- European pharmacists share a convergent ethical core, but the legal force and enforceability of deontological codes diverge across the three regulatory models.
- Professional independence is universally affirmed yet not clearly protected; salary structure, ownership rules, and AI use remain largely outside deontological regulations.

**What are the implications of the main findings?**

- The effectiveness of a deontological framework depends largely on its legal architecture and enforcement mechanism, not on a proclamation of independence
- Professional bodies, rather than EU harmonization, are the realistic vehicle for common minimum standards, particularly for digital practice and AI.

**Abstract:**

**Background:** The modern pharmacist is no longer just a person who prepares medicines, but has become an important health care provider involved in public health, including pharmaceutical care, prevention, chronic disease management and digital health. Such a transition requires the need for professional independence, supported by effective regulation. Deontological codes guarantee this independence and duties but variability in legal frameworks across Europe limits consistent innovation in pharmaceutical care. **Objectives:** The study analyses the deontological framework for pharmacist professional independence across 30 European jurisdictions and evaluates how effectively each national framework protects independence as a precondition for the development of pharmaceutical care, taking into account professional mobility and changes in healthcare system requirements. **Methods:** A comparative analysis examined deontological codes and professional ethics from 27 EU countries, the UK, Norway, and Switzerland. Data were collected using a 14-criterion protocol on topics like legal status, professional independence, conscientious objection, confidentiality, advertising, inter-professional relations, competition, errors, telepharmacy, digitalization, continuity of care, professional development, salary regulation, pharmaceutical services, and incompatibilities. These criteria were organized into five thematic clusters for comparison. **Results:** Three regulation models were identified regarding the legal status of deontological codes: (a) incorporation into primary health legislation, (b) delegation to professional bodies to establish binding corporatist codes, and (c) implementation of soft-law professional standards. While all models share an ethical focus, emphasizing patient priority and confidentiality, they differ considerably in terms of legal bindingness, sanctions, and structural safeguards of professional independence—such as pharmacists’ salaries, ownership rights, and incompatibilities. Telepharmacy is governed by different regulatory frameworks, whereas the application of AI in pharmacy practice has not yet been incorporated into existing deontological codes. **Conclusions:** The current deontological framework affirms professional independence but offers uneven protection; its effectiveness relies more on the legal structure, enforceability, and safeguards than on explicitly enshrined independence. EU-wide harmonization is neither legally feasible nor essential; instead, a shared minimum standard advocated by professional organizations could facilitate European coordination. The digital and AI gap is the most urgent area for deontological updates.

## 1. Introduction

The pharmacy profession and pharmacy practice are grounded in an implicit social contract between society and the practitioner. This social contract grants pharmacists a legal monopoly over the preparation and dispensing of medicines, along with the right to self-regulate the profession through professional bodies. In return, society is assured that competent, certified pharmacists are permitted to practice, that clear, codified ethical conduct is in force, and that public interests are prioritized over commercial interests [1,2]. The normative content of professional conduct is adherence to the four principles of biomedical ethics—autonomy, beneficence, non-maleficence, and justice, which clarify the professional meaning, as the pharmacist is understood not as a supplier of goods or products but as a provider of pharmaceutical care, accountable for patient outcomes [3,4]. Therefore, the deontological code is the instrument through which the profession demonstrates its continued commitment to honor its side of the social contract mentioned above.

The profession of pharmacist has evolved structurally, gradually moving from the role of a ‘drug-maker’ to that of a supplier of essential medical services, centered on the safety and well-being of the patient [5]. This transformation has required a reassessment of ethical fundamentals, which are now no longer just a set of subjective moral norms, but a vital regulatory tool for protecting professional independence from rising trade pressures. Codes of ethics play a critical role in this context. Their aim is to enshrine the fundamental values of the profession in a single document, while promoting ethical behavior and protecting professional status and the right to professional autonomy [6]. The global reference standard is established by the International Pharmaceutical Federation (FIP) through its Code of Ethics for Pharmacists. This document defines the pharmacist–patient relationship as a covenantal relationship based on trust [7]. Furthermore, the Joint FIP/WHO Guidelines on Good Pharmacy Practice (GPP) (2011) stipulate that the primary mission of the pharmacist is to act in the patient’s best interests, regardless of the economic context, thus providing the foundation for globally recognized professional autonomy [8].

The pharmacy has a dual role—as a professional service and a business—creating inherent tensions between these aspects [9]. In recent decades, many European countries have liberalized pharmacy ownership, allowing non-pharmacists to own pharmacies fully or partially [10]. This shift introduces non-pharmacist managers into corporate structures, breaking pharmacists’ monopoly on management. When ownership, control, and professional judgment are separated, professional independence is no longer inherently protected and becomes vulnerable to commercial influences. Pharmacists must therefore balance their professional responsibilities with the organizational demands placed on them. This situation highlights the importance of deontological codes, which serve as a protective framework beyond mere practice standards, and their legal structure should reflect this reality [9]. 

At the EU level, the legal basis is Directive 2005/36/EC on the recognition of professional qualifications. Although it focuses on the harmonization of competences (Art. 44), it lays down minimum training requirements that enable the European pharmacist to act as a public health expert [11]. This ethical dimension has been reinforced by the provisions of the Court of Justice of the European Union (CJEU) in cases such as Joined Cases C-171/07 and C-172/07, which held that the pharmacy is not a mere commercial space but a public health institution, thus justifying strict ethical and professional ownership restrictions to protect the independence of the professional decision [12].

The Pharmaceutical Group of the European Union (PGEU), the EU-level body representing the national professional bodies of community pharmacists, advocates for the profession’s public health role in European policy [13]. Recent research highlights the need for a common ethical framework also in the context of digitalization; For example, the 2025 proposals for a European ethical framework in e-Pharmacy insist on data protection and the maintenance of online care standards [14]. At the same time, recent discussions on the conscience clause (2024) show that, although there is a convergence toward the values of autonomy and dignity of the patient, their application in the national codes varies significantly [15,16]. Although the pillars of regulation, from the FIP Covenant to EU Directives, are common to the European Union, United Kingdom, Switzerland, and Norway, their transposition into national deontological codes reflects a complex diversity [17].

The published comparative studies approached this diversity of codes and regulations selectively. Raimundo and Cavaco compared codes from a culturally diverse set of 3 European countries using the lens of their professional statements and ethical content [6]. Another study by Romanian researchers examined individual themes in depth—the conscience clause across EU codes and the ethical framework in the e-pharmacy field [14,15]. A comprehensive analysis that treats codes jurisdiction by jurisdiction, their legal architecture, and, especially, the protection of professional independence is needed, and this study aims to fill this gap. To the best of our knowledge, this is the first systematic comparison of the deontological frameworks governing pharmacists across 30 European jurisdictions (27 EU Member States, the United Kingdom, Norway, and Switzerland) using a structured 14-criterion grid, organized into five thematic clusters. Instead of focusing solely on convergence, our study aims to assess how effectively each framework protects professional independence, on which the integrity of pharmaceutical care ultimately rests.

## 2. Materials and Methods

In preparing this study, codes of ethics and similar regulatory acts from 27 European Union member states, as well as from Switzerland, Norway, and the United Kingdom, were analyzed; these were collected and consulted from the websites of professional associations or the relevant competent authorities.

Ethical standards for pharmacy practice were identified through qualitative document analysis of relevant texts [18]. A comparative approach was used to demonstrate similarities and differences among documents related to professional ethics, in order to map the diversity of national frameworks. Reporting followed the Standards for Reporting Qualitative Research (SRQR; Table S2).

The results of the textual analysis were categorized and interpreted in order to extract information about pharmacists’ deontological standards. The comparative legal research employed a logical method to examine the structure and content of the relevant normative acts. The critical method was used to evaluate the extent to which existing deontological frameworks are sufficient for the professional role of the modern pharmacist, particularly with respect to safeguarding professional independence. In particular, the analysis was structured around 14 main criteria specified at the beginning of data collection. These are presented in Table 1 grouped into five thematic clusters.

Two authors (MCC, ASA) developed the 14 criteria before data collection, based on the content of the Romanian deontological code for pharmacists and themes identified by researchers in an ongoing study of pharmaceutical malpractice. The 14-criteria framework used in this study was not validated nor assessed for content validity. The study maintained consistency across extractors through a fixed template and source-level verification. The five thematic clusters presented in Table 1 were not part of the extraction instrument but were derived inductively post-extraction to provide an organizing scheme for presentation and analysis. Ten pharmacists in residency training initially extracted the data between January and February 2026 as part of a project. Each pharmacist in residency training was assigned to three jurisdictions. The extraction used a standard template listing the 14 criteria and followed verbal instructions on the work procedure. For each criterion, they recorded the findings, and the sources were also listed. Documents were included if issued by a national authority or pharmacy professional body and addressed one or more of the 14 criteria. The main focus was the national deontological or ethical code for pharmacists. If absent or incomplete, legislation on pharmacy organization or practice was used. The current version at the time of extraction was considered, with no language or adoption date restrictions. The only exception was criterion 12 (remuneration), for which other sources were used, as most deontological codes do not include such provisions. For documents available only in national languages, a machine translation was used (Google Translate, DeepL). Verification of the extracted data was performed by two authors in April-June 2026 and again by three authors in August. Verification at this stage was conducted at the source level—each cited source was checked for existence, correct identification, and coverage of the criterion to which it had been assigned. Since extraction recorded legal provisions rather than interpretative judgments, discrepancies took the form of incorrect or insufficient source attribution rather than disagreements about meaning and were resolved by correction. All 30 jurisdictions were checked twice. The three-model classification of Section 3.1 was developed inductively from the analyzed material and validated by one of the authors holding a doctorate in law (SBB), not involved in the extraction phase. In preparation of the full 30 × 14 matrix, all extracted data were re-verified at the content level and reported in the Supplementary Materials as Table S1. Each extracted data point was checked against its source, key terms were verified, and source texts were re-translated using Reverso—a different engine than initially used.

The authors used Generative AI (Claude Opus 4.8, Anthropic, San Francisco, CA, USA) for translation of author-drafted text from Romanian to English, language editing and structural organization. It was not used to generate, extract or interpret data, all of which remained the responsibility of the authors.

## 3. Results

### 3.1. The Legal Architecture and Status of Deontological Codes

**Figure 1:**
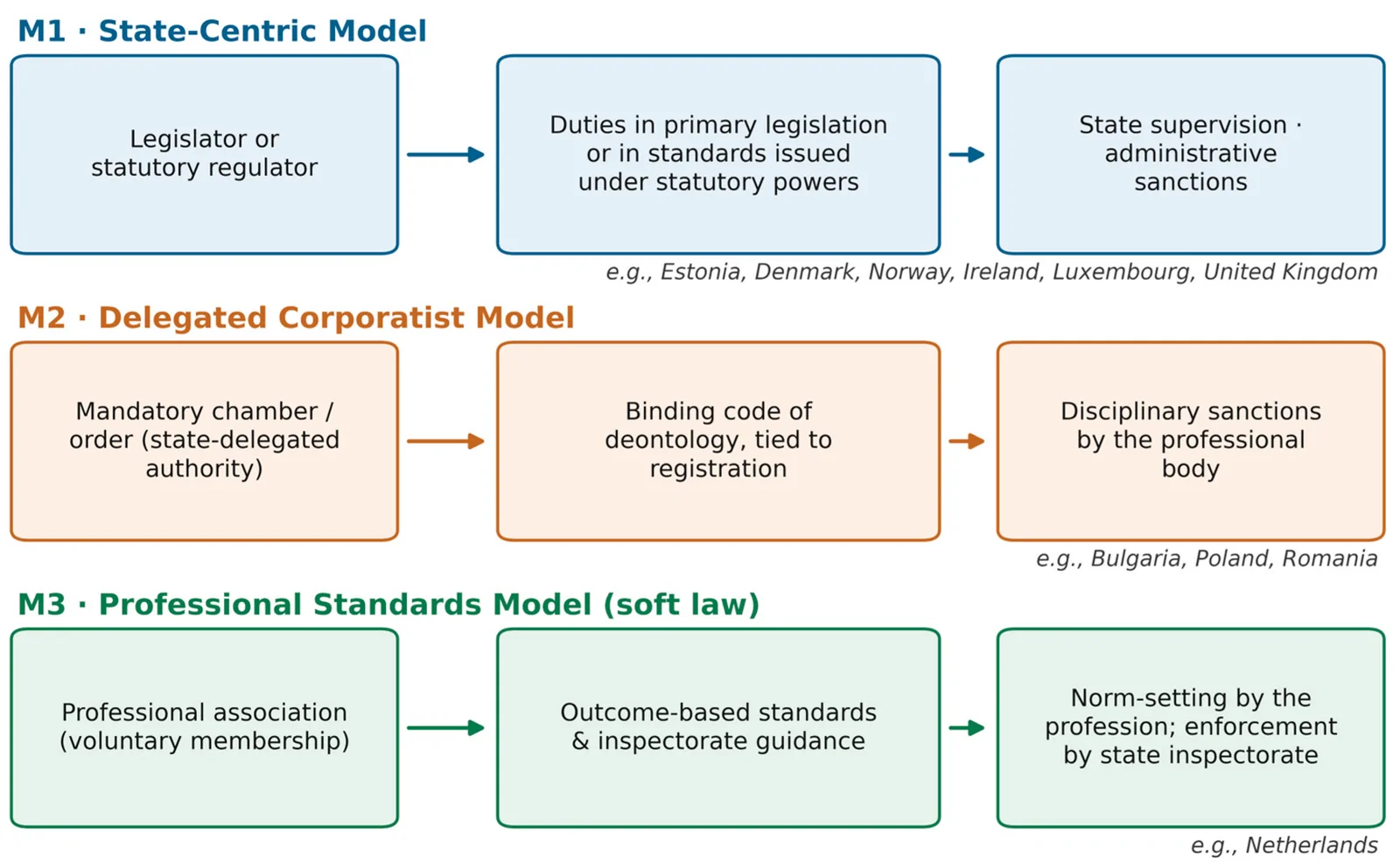
The three deontological regulatory models identified across the 30 jurisdictions: norm-setting authority, legal instrument, and enforcement mechanism.

Across the 30 European jurisdictions examined, the legal status and official nomenclature of the pharmacist’s ethical instrument exhibit strong convergence in ethical content, despite marked diversity in their legal form [6,14]. Most systems explicitly align their national instruments with the global benchmarks set by the International Pharmaceutical Federation (FIP) Statement of Professional Standards (also known as the Codes of Ethics for Pharmacists) and the Joint FIP/WHO Guidelines on Good Pharmacy Practice, which define core duties such as patient welfare, professional independence, and confidentiality [7,8]. At the European Union level, Directive 2005/36/EC on the recognition of professional qualifications underpins mobility by establishing automatic recognition for pharmacists who meet the harmonized minimum training conditions, while leaving professional ethics and supervision primarily to national regulation [11]. Based on this common foundation, three main legal models emerge across the continent, for example, the State-Centric Model, the Delegated Corporatist Model, and the Professional Standards Model (also known as Soft Law). Figure 1 summarizes these three models in terms of the authority that sets the norm, the instrument through which it is expressed, and the mechanism through which it is enforced.

#### 3.1.1. The State-Centric Model (M1)

In this model, ethical duties are embedded directly in primary legislation and sectoral regulations rather than within a standalone “professional code”. Estonia can be treated as a state-centric example, where pharmacist obligations and services appear to be anchored principally in the Medicinal Products Act and the Health Services Organization Act and related implementing rules, rather than in a separate professional ethics code adopted by a chamber [17]. Denmark and Norway follow the same logic, with pharmacist duties anchored in the national Pharmacy Act [19,20]. The model also covers jurisdictions where the ethical instrument is issued by a statutory regulator acting under direct state authority rather than by a self-governing chamber, as in Ireland and Luxembourg [21,22,23]. In the United Kingdom, Article 48 of the Pharmacy Order 2010 empowers the General Pharmaceutical Council (GPhC) to establish binding standards relating to the conduct, ethics, and performance of pharmacists, which are firmly grounded in national pharmacy legislation. Still, they are framed as outcome-based “standards for pharmacy professionals” rather than article-based codes [24,25].

#### 3.1.2. The Delegated Corporatist Model (M2)

This framework is the dominant pattern in Europe. The state delegates norm-setting power to a mandatory public-law authority (order, chamber, or council), and explicitly empowers it to adopt a binding code of ethics. The pharmacist has no right to practice if he is not registered.

In Central and Eastern Europe, some countries like Bulgaria, Croatia, Romania, Poland, and the Czech Republic use deontological codes adopted by national chambers that possess disciplinary competencies. For example, Bulgaria operates through the Bulgarian Pharmaceutical Union (BPU), a public-law professional body established under the Law on the Professional Organization of Master Pharmacists, in which membership is compulsory for every practicing pharmacist as a prerequisite for professional registration. The BPU autonomously adopts and enforces the Code of Professional Ethics of the Master Pharmacist, exercising its own disciplinary authority to impose sanctions for violations, including fines collected directly by the regional pharmaceutical collegia [26,27].

The Czech Code of Ethics of Pharmacists and Croatia’s Code of Ethics and Deontology of Pharmacists, grounded in the Pharmacy Act and the Act on Healthcare, play a similar role as compulsory professional legal instruments attached to licensure [28,29,30,31]. Poland’s Kodeks Etyki Farmaceuty Rzeczypospolitej Polskiej and Romania’s Codul Deontologic al Farmacistului likewise function as formal deontological codes adopted by national professional chambers with explicit disciplinary competences [32,33].

#### 3.1.3. The Professional Standards Model—Soft Law (M3)

A third group of governance relies on standards, inspectorate guidance, or voluntary professional norms rather than article-based ethical codes.

At the same time, Nordic and Benelux countries emphasize inspectorate guidance and professional standards, in line with broader trends highlighted by the World Health Organization Regional Office for Europe and the PGEU Professional Standards and Ethics in Pharmacy [17,34].

The Netherlands operates without a mandatory professional chamber. The voluntary KNMP (Koninklijke Nederlandse Maatschappij ter bevordering der Pharmacie), legally a private association, develops professional norms through its Code of Pharmacists, which the state inspectorate IGJ then adopts as enforceable field standards (veldnormen) for administrative inspection and disciplinary proceedings before the Central Disciplinary Board for Healthcare. This model thus separates norm-setting, which remains with the profession, from enforcement, which is exclusively reserved for state authorities, distinguishing it from the corporatist chamber systems dominant across continental Europe [35,36].

Overall, the first criterion indicates that, despite the use of different legal vehicles, ranging from primary statutes to corporatist codes and soft law standards, national instruments almost always present themselves as formal ethical frameworks tied to registration and discipline, and are conceptually aligned with the FIP/WHO global standards. Table 2 shows the distribution across the 30 jurisdictions. The most prevalent model is the delegated corporatist model (M2) with 16 jurisdictions (53.3%), followed by the state-centric model (M1) with 8 (26.7%) and the professional standards model (M3) with 6 (20.0%). Regarding legal force, 25 instruments (83.3%) are binding and enforceable through disciplinary, administrative or penal mechanisms, while 5 (16.7%) are voluntary professional guidance.

### 3.2. Professional Independence

The principle of professional independence constitutes the ethical core of modern pharmacy practice, but its true efficacy depends on how it is put into practice in the face of both internal moral dilemmas and external market forces. More precisely, the pharmacist’s independence is continuously balanced between the intersection of clinical responsibilities and personal convictions—particularly concerning conscientious objection to dispensing and is simultaneously insulated by compensation systems explicitly designed to offset commercial pressures. An analysis of these three dimensions reveals how different jurisdictions, in practice, safeguard the integrity of professional decision-making.

#### 3.2.1. Safeguards for Clinical Autonomy

Protecting the pharmacist’s clinical autonomy against commercial pressures is a foundation stone of European pharmaceutical regulation. Across the 30 jurisdictions, rules governing professional independence and conflicts of interest fall into three broad regulatory models, for example: statutory controls, corporatist deontological codes, and general professional standard obligations.

- *Statutory Anchoring with Binding Codes*. Independence is defined directly in pharmacy or health legislation and reinforced by a deontological code that has clear legal consequences for licensing and discipline. The most important details related to Bulgaria, Croatia, Romania, and Luxembourg are highlighted in the following paragraphs.

Bulgaria links the Medicinal Products in Human Medicine Act and the Bulgarian Pharmaceutical Union’s Code of Professional Ethics for pharmacists, so that commercial influence, collusion, and other conflicts of interest can trigger both administrative and professional sanctions [26].

Croatia follows a similar pattern: the Pharmacy Act and the Croatian Chamber of Pharmacists Code of Ethics and Deontology of Pharmacists jointly require pharmacists to prioritize patient safety, report and document errors, and avoid financial arrangements that might compromise impartiality [29,30].

Romania’s Deontological Code of Pharmacists (2009) explicitly frames independence as a duty towards the patient and public health, prohibiting unfair competition and any benefits that could influence professional judgment [32].

Luxembourg’s pharmacy law and Code de Déontologie des Pharmaciens similarly demand that pharmacists act in the public interest and avoid conduct that would undermine trust in the profession [51,60].

- *Corporatist Professional Orders*. In this model, orders with quasi-legal deontological codes, in a second group of countries, where strong professional orders adopt comprehensive ethical codes with quasi-legal force, backed by sectoral legislation but implemented mainly through disciplinary mechanisms of the order.

In countries like Ireland, Malta, Portugal, Spain, and Switzerland, where national orders’ codes articulate independence as a core professional value, restrict advertising and financial incentives, and define “incompatibilities” such as business arrangements that could compromise clinical judgment [6,14,59]. In Switzerland, for instance, the pharmaSuisse: Code de déontologie de la Société Suisse des Pharmaciens emphasizes impartial counseling and avoidance of commercial pressures, complementing the statutory regulation of pharmacy ownership [59].

- *State-Centered General Obligations*: A third model, seen prominently in Estonia and several Nordic/Anglo-Saxon systems, relies less on a binding professional code and more on general duties in health professional and medicines legislation [14,40].

In Estonia, there is no single deontological code with autonomous disciplinary force; instead, pharmacists’ conduct is regulated through the Health Services Organization Act and the Medicinal Products Act, with sanctions imposed administratively or judicially rather than corporatively [40]. These laws, together with detailed advertising controls, require therapeutic impartiality, prohibit collusion with prescribers, and limit commercial practices that might distort professional decision making [14,40].

Across all three models, recent comparative work on conscience clauses and digitalisation shows a convergent expectation that pharmacists resist commercial pressures and protect autonomous clinical judgment, but with significant variation in how strongly and precisely conflicts of interest are regulated [6,14,34].

#### 3.2.2. Conscientious Objection and the Refusal to Dispense:

In European pharmacy law, conscientious objection and the refusal to dispense are governed by a convergent regulatory logic that prioritizes patient safety and continuous access to medicines over individual moral autonomy.

A comparative analysis of European Union pharmacist regulation reveals that most jurisdictions reject broad, open-ended rights to refuse dispensing on moral or religious grounds, treating refusal instead as an exceptional measure tied to legal and clinical criteria [6,14]. Systematic document analysis of pharmacist codes of ethics confirms this pattern, with refusal clauses embedded in sections on professional responsibility and patient protection rather than in provisions on freedom of conscience [6,16,34].

European professional standards and ethical guidance from PGEU and FIP reinforce this orientation, stressing that pharmacists may decline to dispense only where medicines are unsafe, prescriptions are invalid, or legal conditions are unsatisfied, and that continuity of care must be safeguarded [7,8,34]. Furthermore, Directive 2005/36/EC includes these expectations within the mutual-recognition framework for professional qualifications, presuming that mobile pharmacists will respect host country rules on patient safety and access to therapy [11].

Across the 30 jurisdictions, the overarching trend is convergence towards treating refusal to dispense as a tightly regulated professional safety mechanism rather than as an expression of individual moral autonomy [8,15,16]. The predominant model—safety-based mandatory refusal is established in countries such as Germany, Sweden, France, Spain, Romania, and Belgium, where it legally obliges pharmacists to refuse dispensing in cases of therapeutic risk, invalid prescriptions, or legal non-compliance. Still, it explicitly rejects broad moral or religious objections that would leave patients without medication [6,7,8,15,16,44]. In Romania, pharmacists play an essential role in ensuring the safety of patients, in counseling patients about the potential benefits and risks of their treatments, and they must stay up to date on the latest information about new treatments [61]. In this context, assessing treatment safety is considered a mandatory clinical obligation, not an optional choice [15,16].

A second approach, professional autonomy-framed refusal, was observed in jurisdictions like Latvia, Poland, Slovenia, and Ireland. These codes affirm that pharmacists act according to conscience and professional judgment, but do not codify a general right to conscientious objection or a standardized procedure for invoking it. Pharmacists act according to their professional judgment, allowing refusal when prescriptions contravene professional standards, provided the decision is documented and does not compromise continuity of care [6,15,16,33,56,59].

A minority approach—patient-centered accommodation model represented by the United Kingdom (GPhC standards), alongside certain Nordic and Swiss models, where guidance acknowledges that pharmacists may decline to participate in certain services for deeply held beliefs, but imposes stringent duties to ensure timely alternative access. Also, ethical guidelines recognize potential conflicts of conscience but state clearly that patient welfare, non-discrimination, and continuity of therapy override individual preference in the dispensing context [6,7,15,16,34,59,62,63].

Overall, the comparative landscape highlights a continent-wide convergence toward treating the refusal to dispense medications as a strictly regulated clinical safety mechanism, rather than as an unlimited personal right [15,16]. Because the vast majority of European systems limit refusal to objective legal and therapeutic reasons, these clinical ethical standards do not constitute structural barriers to pharmacists’ mobility within the EU’s mutual recognition system [11,15,16]. The persistence of soft-law-based accommodation in a few jurisdictions indicates an ethical and regulatory frontier where future European-level guidance could further harmonize expectations while respecting legitimate diversity in moral outlooks [11,14,15,16].

#### 3.2.3. Remuneration Models as Ethical Protections

The second aspect of this topic is represented by the remuneration model. The economic dimension of pharmacy practice raises fundamental ethical questions regarding how remuneration structures influence professional decision-making. European frameworks manage this challenge through two primary approaches:

- *The Fixed/Professional Fee Model*: Countries like Finland and Hungary tie the pharmacist’s income to the professional act itself, generating relatively uniform salary levels and insulating clinical judgment from market dynamics.

Finland exemplifies this approach: salaries are established through collective bargaining between the Finnish Pharmacists Association (Suomen Farmasialiitto) and the Employers Association of Finnish Pharmacies (Apta), with standardized pay scales structured according to professional qualifications and work experience [64,65].

In Hungary, in community pharmacies, salaries are set through individual employment contracts entered into with the employer (the pharmacy), in accordance with the mandatory minimum wage thresholds established by the national guaranteed minimum wage for holders of higher education degrees. Pharmacy earnings are significantly influenced by regulated profit margins and by reimbursement and service payments provided by the National Health Insurance Fund Administration (NHIFA). This mechanism ensures compensation for pharmacies and pharmaceutical services. [66,67,68].

- *The Commercial/Commission Model and Regulatory Interventions*: Where financial incentives are tied to product sales, systems implement strict deontological protections.

For example, Germany addresses the risk through the Fremdbesitzverbot rule, which prohibits non-pharmacist ownership of pharmacies to prevent corporate chains from imposing commercial targets [69]. Similarly, Ireland maintains transparent public salary grids for the public sector (HSE) while leaving private sector compensation to market negotiation [70]. Denmark remunerates specialized services (medication reviews, inhaler training, new medication initiation programs) separately from the dispensing act through regional or national funding [19].

Furthermore, division—the fee-splitting between prescribers and dispensers—is explicitly prohibited across multiple jurisdictions. Hungarian law forbids practitioners from both prescribing and dispensing medications to their own patients, establishing a legal separation between the medical and pharmaceutical acts [71]. Dutch regulations prohibit pharmacists from holding interests in wholesale distribution or manufacturing companies, ensuring therapeutic impartiality [53]. Finnish legislation restricts the simultaneous practice of medicine and pharmacy to prevent profit generation from self-generated prescriptions [72].

The prohibition of commercial bonuses that could compromise objectivity appears consistently in stricter deontological frameworks. The Netherlands maintains that acceptance of financial benefits from the pharmaceutical industry is strictly forbidden, with any conflict of interest considered incompatible with professional practice [36]. Ireland requires public salary transparency in the HSE sector, while maintaining that health priorities must override commercial interests [73]. The Court of Justice of the European Union reinforced this paradigm in Joined Cases C-171/07 and C-172/07, ruling that pharmacies constitute healthcare institutions rather than commercial enterprises, thereby justifying deontological restrictions on commercial activities [12]. The Pharmaceutical Group of the European Union (PGEU) has consistently maintained that professional standards must shield pharmacists from commercial pressures that could undermine patient care [29].

**Figure 2:**
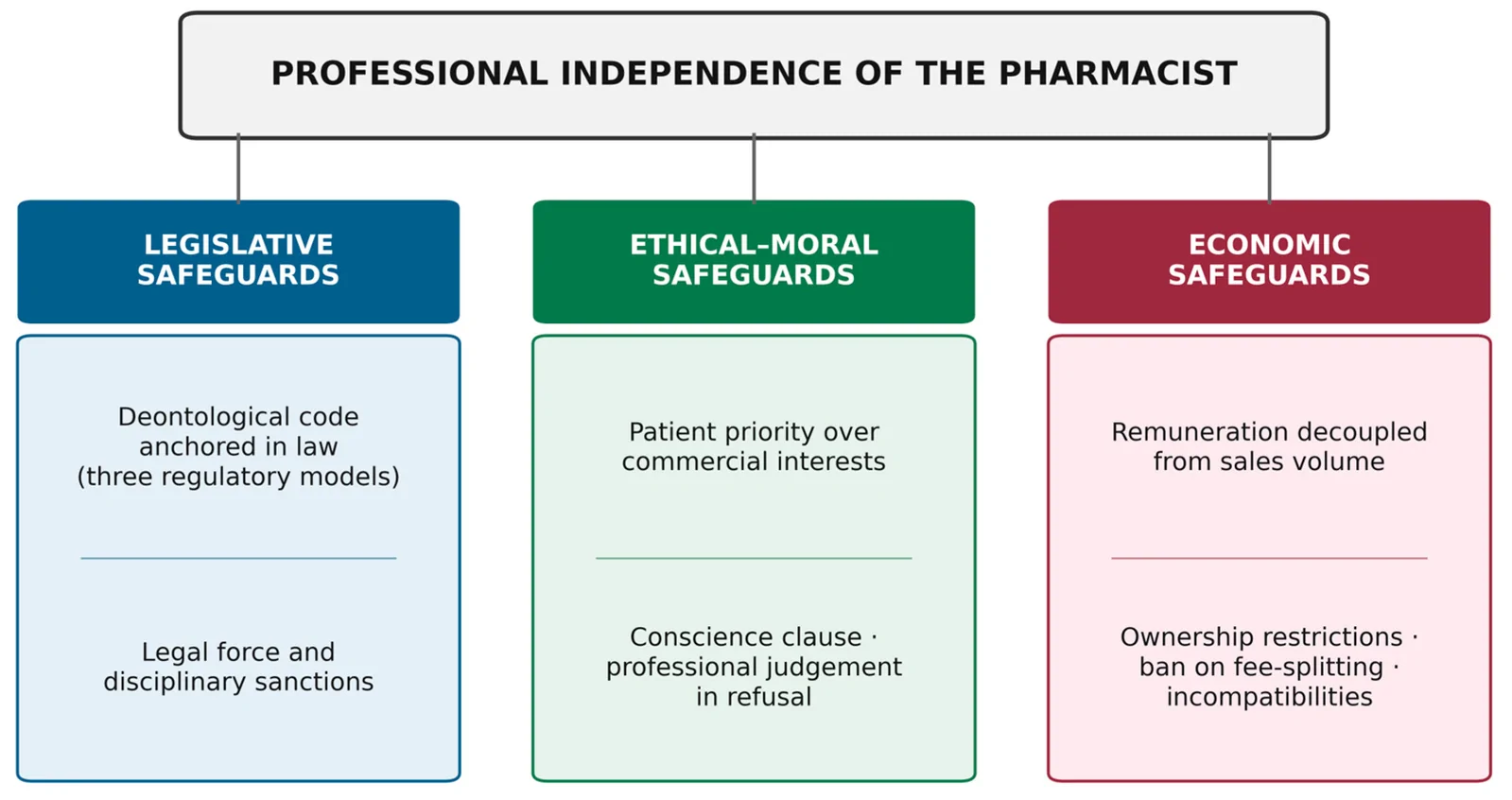
The three dimensions of safeguards for pharmacist professional independence identified across the deontological frameworks analyzed. The three regulatory models are presented in Figure 1.

The aspects mentioned above are structurally shown in Figure 2.

### 3.3. The Pharmacist-Patient Relationship and Clinical Responsibility

The pharmacist-patient relationship has fundamentally transitioned from a transactional interaction into a clinical partnership centered on patient safety and comprehensive care. This shift is structurally supported by rigorous frameworks governing data confidentiality, the management of professional errors, the assurance of continuous care, and the evolution of remunerated pharmaceutical services.

#### 3.3.1. Professional Secrecy and Data Confidentiality

Across the 30 European countries examined, professional secrecy and patient confidentiality emerge as universal ethical pillars in pharmacy, yet the concrete legal bases, enforcement mechanisms, and digital safeguards show marked cross-national variation [6].

Comparative analysis of pharmacy codes and national frameworks reveals a first group of states where confidentiality is rooted directly in criminal law, with breach of professional secrecy constituting a specific offense and the duty persisting beyond patient death or cessation of practice [14].

- *Penal and Perpetual Protection*: In this group, exemplified by Germany and several Central European jurisdictions, secrecy covers everything the pharmacist learns about the patient, while narrowly framed exceptions allow disclosure only with patient consent, to protect a higher legal interest, or to comply with mandatory reporting duties for infectious diseases and serious adverse reactions [7,74].

Furthermore, Spain, Switzerland, and Norway add perpetual protection under penal provisions to strict limits on disclosure, generally permitting access to confidential information only based on a court order, explicit statutory mandate, or imminent risk to public health [8,20,23,59].

- *Digital safeguards:* In highly digitalized Nordic and Baltic systems, including Estonia and Norway, this strong legal protection is operationalized through national e-health platforms in which pharmacists may access electronic health records solely for dispensing or medication review; every access is logged, and unjustified consultations trigger disciplinary or even criminal consequences [20,41].

Estonia illustrates this model clearly: since professional secrecy is protected by medical data legislation and a dedicated health information system law, pharmacists may consult patient data only to supply treatment, and any unjustified access to records is automatically recorded and subject to sanction [40,41].

- *Statutory and Registry Data*: A large cluster depends on secrecy primarily in health professional statutes and data protection law, with the code of ethics reiterating that confidentiality covers all diagnostic, therapeutic, and medication.

Several Central and Eastern European codes, including those of Bulgaria, Croatia, Slovakia, Poland, and neighboring states, expressly require confidentiality for everything learned in the exercise of the profession and for data contained in pharmacy or professional registers, while placing detailed advertising or competition rules in separate legislation [26,29,33,35]. Southern and island systems like Malta, Cyprus, and Ireland similarly impose a legal and ethical obligation of secrecy for all sensitive information obtained in practice, maintain confidentiality until explicit patient consent is given, and allow disclosure without consent only to protect life or comply with court or statutory requirements [73,75,76].

The Slovak framework, for example, imposes an explicit obligation of professional secrecy over information obtained during practice and over registry data, underlining that disclosure is acceptable only where required by law or necessary to safeguard patient welfare [77].

#### 3.3.2. Management of Professional Error

Professional error is treated across Europe as a patient safety failure that activates verification duties, documentation, counseling, and escalation when a preventable dispensing mistake causes harm, rather than remaining a private ethical concern alone [6].

- *Layered Liability and No-Fault Compensation*: This is the common model because civil compensation, disciplinary review, and, in some systems, penal exposure coexist when unsafe practice or recurring neglect shows that one remedy is insufficient for everyone involved [14].

Finland is the clearest outlier, because incident reporting is central, compensation is routed through the patient insurance center, and the patient does not need to prove individual pharmacist guilt before payment is made directly [7,78]. That model is still not permissive, because serious or repeated failures permit inspection, practice restriction, or withdrawal of rights when the workflow continues to generate unsafe outcomes and the pharmacy itself becomes a public risk [8].

Estonia sits at the opposite pole, because the pharmacist remains personally accountable for correct dispensing, prescription checking, and therapeutic safety. At the same time, electronic records preserve traceability for legal review by authorities in the future. In that setting, malpractice is direct legal responsibility, and the state can translate an unsafe act into civil, administrative, or penal consequences when gravity demands it, and the record supports intervention clearly [40,79].

- *Accountability and Deontic Strictness*: The Czech and Slovak codes preserve a more classical deontic style because they emphasize personal responsibility, truthful information, and refusal of unsafe or unlawful action as ethical duties rather than optional ideals alone [28,55].

The Czech code is especially strict since it gives the code disciplinary force and rejects delegation to unqualified staff, which narrows excuses for avoidable mistakes in everyday pharmacy practice across dispensing work itself [28]. Slovakia is more procedural because the ethical code is annexed to the profession law, and online sales are handled mainly through medicines legislation, notification duties, and licensing conditions [77,80]. Its system shows a thinner malpractice architecture than the northern systems, yet continuing education remains explicit, suggesting prevention is emphasized more clearly than sanction design, and competence maintenance is the main barrier in practice [81].

Furthermore, Slovenia combines statutory rules, chamber ethics, state oversight, and formal reporting, so the error response is institutionally distributed rather than left to a single actor or a single disciplinary path alone entirely [56]. It also separates ordinary error from conflict-of-interest-driven misconduct, which means economic steering, patient redirection, and advantageous benefits are treated as stronger integrity breaches than a routine dispensing mistake alone today [82].

Across the broader map, the shared European idea is that the pharmacist is a clinical gatekeeper obliged to question prescriptions, prevent harm, and document what was done or missed carefully in practice generally. In modern clinical pharmacotherapy, the prevention of some adverse iatrogenic effects is increasingly based on the pharmacist’s clinical ability to identify additive risk related to chemical drug structure, for example, severe neuropsychiatric adverse reactions [83]. The most important divergence is the institutional answer after harm because some systems prioritize compensation and learning, others fault and sanction, and a few combine both with unusually sharp regulatory tracing for all parties. Taken together, the comparative map shows a continental convergence toward patient safety, but a persistent divergence in how blame is translated into compensation, sanction, or prevention across modern pharmacy law today [84,85].

#### 3.3.3. Continuity of Pharmaceutical Care

The European regulatory landscape for pharmacy duty services reveals two fundamentally divergent models for ensuring population access to medicines outside regular business hours, such as the mandatory public service and the market-adjusted volunteer and exceptional models.

- *The Mandatory Public Service Model*: This model treats pharmaceutical continuity as a non-negotiable state obligation.

Denmark operates the most structurally developed version: the state establishes duty shift schedules to ensure territorial coverage, and a financial compensation mechanism redistributes operational costs so that pharmacies providing shifts receive subsidies funded by those that do not [86]. Germany delegates this function to the Regional Chamber of Pharmacists, which produces an annual rotation calendar for all night and Sunday shifts within its jurisdiction; a legally fixed emergency [87]. Estonia organizes permanence at the regional level under direct coordination of health authorities, treating access to medicines as an essential public service where each geographic zone is permanently covered, and the pharmacy has no discretionary power over participation [79,88]. Slovenia reflects this architecture thus: local authorities jointly plan mandatory permanence programs with public providers, designated pharmacies must dispense outside standard hours, and a nationally regulated emergency fee may be applied [56,89].

- *The Market-Adjusted Volunteer and Exceptional Models*: This model assigns continuity obligations primarily to the profession itself.

Finland establishes operating hours through the Medicines Agency; Fimea may impose conditions regarding the operating hours of pharmacies, as part of their operating license, when necessary to ensure access to medication [90]. Austria requires on-call pharmacy service on a rotation basis under pharmacy legislation, but pharmacists retain an independent emergency dispensing right: they may supply medicines without a prescription in minimum quantities in genuine emergencies [91]. Belgium organizes duty services through local rotation between pharmacies [33]. Irish system, involving coordination with the Pharmaceutical Society of Ireland and continuity of patient care in emergency situations, stipulates that pharmacists, under defined conditions, may supply certain prescription-only medicines without a prescription [92,93].

Cyprus occupies a singular position: there is no formal on-call duty system at all. Pharmacies operate according to a fixed schedule with a midday lunch break; most are closed on Sundays, and rotation extends only until midnight. A telephone consultation service represents the sole after-hours clinical contact option [75,94]. The United Kingdom follows a similarly minimal pattern with very few genuine 24 h pharmacies; most are open until midnight, and online delivery with next-day arrival serves as the principal after-hours access mechanism [25,95]. Italy presents a distinct organizational logic: continuity of pharmaceutical care is satisfied through regulated emergency dispensing in genuine necessity situations and through regulated home delivery when the original prescription has been transmitted to the pharmacy, both governed by the deontological code and the National Health Service framework [48].

The structural tension across all models concerns who bears the financial burden of after-hours coverage. Nordic and Central European mandatory models distribute costs through subsidies or institutional budgets. Southern and Atlantic models leave the burden more directly on the participating pharmacy, with rotation serving as the primary equity mechanism. The emergency dispensing right, present in Austria, Italy, and Malta, represents the most permissive safety net across all frameworks.

Furthermore, independent locum practice can function as a practical mechanism for sustaining continuity of care, particularly in rural or understaffed jurisdictions, by allowing pharmacists to provide after-hours and temporary coverage without being organizationally subordinated to a single dispensing business [96].

#### 3.3.4. The Shift Toward Pharmaceutical Services

The shift from a product-centered to a service-oriented practice represents one of the most consequential transformations in European healthcare legislation over the past decade. Two fundamentally different models coexist across the continent, and the way services are remunerated shapes the very identity of the profession.

- *The Product-Oriented Model*: This model ties the pharmacist’s income to the margin generated by the medication itself, with clinical activities bundled into the dispensing act without separate compensation.

Estonia exemplifies this approach: pharmaceutical services such as counseling, therapy verification, and monitoring are considered integral to the medication distribution and compensation system, with pharmacies receiving remuneration through regulated markups and health insurer reimbursement mechanisms [40]. Hungary follows a comparable logic: pharmaceutical services are not organized as individually billed clinical packages but are integrated into the compensated dispensing act and the pharmacy’s contractual obligations toward the national insurance fund (adherence monitoring, generic substitution, therapy validation) [68,71]. Greece represents a third variant of the product model, where clinical services remain poorly developed and weakly compensated, with extended counseling or therapy monitoring typically included in the dispensing act without separate remuneration [46].

- *The Service-Oriented Model* decouples the professional fee from the price of the medicine, treating the pharmacist as a provider of measurable clinical interventions.

Germany launched the most ambitious reform in 2022 with Pharmazeutische Dienstleistungen (pDL): services such as polypharmacy medication review, instruction on correct inhalation technique in asthma or COPD, and blood pressure measurement are paid directly by insurance funds to pharmacies, with monthly earnings ranging for management or specialized roles [97]. Ireland operates a mixed funding model where services, including vaccination (flu, COVID, and others), screening, smoking cessation, and medication therapy monitoring, are financed either through public funds via the Health Service Executive (HSE), directly by the patient, or through combined mechanisms, depending on service type and patient eligibility [73]. Denmark covers services such as “Medical Check-up” (medication review), inhaler training, and support programs for new users of chronic medications, all paid directly to pharmacies by regions or the state [19].

Among the most striking national outliers, Belgium introduced pharmacist-administered vaccination as a defined pharmaceutical service, with reimbursement varying by vaccine type: some are fully covered by public health insurance, while others require patient co-payment [98]. Italy represents a particularly forward-looking case in the evolution of pharmacy services supported by annual government allocation. New pharmacy services through the national budget now include participation in integrated home care, and services such as antibiotic resistance testing, telemedicine, and hepatitis C screening have been formalized through legislation (Law 182/2025) [99]. Finland integrates most services legally into the “Patient Care Plan,” making them non-optional and non-commercially driven, with funding split between the national health insurer (Kela), municipalities, and patient co-payments for specialized services [72,100]. In Austria, a community pharmacist can provide basic testing (blood pressure, blood sugar) and a monitoring function while maintaining strict safety conditions for home delivery for immobilized patients. Furthermore, Austrian pharmacy vaccination programs provide vaccines and vaccination counseling, although, in general, pharmacists are not authorized to administer vaccines under current pharmacy laws. [37].

In the Romanian system, most clinical services remain unpaid or financially unregulated, with basic pharmaceutical services limited to dispensing, patient counseling, compounding, and pharmacovigilance, while supplementary services such as polypharmacy monitoring and chronic disease management exist on paper but lack structured reimbursement mechanisms [25].

The figure below illustrates the three areas of regulation discussed earlier (Table 3).

### 3.4. The Commercial Environment, Competition, and Professional Incompatibilities

The European pharmaceutical sector thoroughly regulates the pharmacist’s interaction with the commercial environment to prevent market dynamics from subordinating clinical judgment. This is achieved by imposing ethical barriers between prescribing and dispensing, severely restricting advertising, regulating inter-pharmacy competition, and defining strict professional incompatibilities.

#### 3.4.1. Pharmacy Advertising and Self-Promotion

Across the continent, advertising is heavily restricted to project the image of the pharmacy as a public health institution rather than a retail enterprise.

- *Statutory and Absolute Restrictions*: This category included various countries, like Germany, Italy, Slovenia, Poland, Estonia, and Romania.

Germany’s Heilmittelwerbegesetz (HWG) anchors the restrictive philosophy by prohibiting comparative pharmacy advertising, miraculous cure promises, and the offering of gifts or prizes for prescription medicines, thereby legally compelling the pharmacist to project the image of a health provider rather than a merchant [101]. Italy extends this protective logic through the FOFI Code of Ethics, which forbids pharmacists from accepting pharmacy promotion via advertising and explicitly bans prize contests involving pharmaceutical products, restrictions further entrenched in legislative Decree 223/2006 [48,102]. Slovenia’s Code of Pharmaceutical Ethics and the Medicinal Products Act confine advertising to a strictly professional framework, banning financial incentives, conditional discounts on prescription medicines, and any promotional practice that could induce irrational drug use or influence treatment choice [56]. Poland’s Pharmaceutical Law, notably Article 94a, imposes severe legal restrictions on pharmacy advertising, a stance the Court of Justice of the European Union has affirmed as proportionate in recent case-law [12,103]. Estonia’s Medicinal Products Act regulations bar the promotion of prescription medicines to the public and require that over-the-counter information remain objectively factual, free of financial inducements or commercially motivated recommendations [40]. Romania’s Law 95/2006 prohibits prescription drug advertising to the public, and for those classified as psychotropic substances or narcotics. Advertising aimed at the general public only for those medicines that, by their composition and scope, are intended to be used without the involvement of a doctor for diagnosis, prescription, or treatment monitoring [104].

- *Nordic and Objective Paradigms*: Finland’s Medicines Act and Fimea oversight add granular commercial prohibitions by explicitly forbidding marketing techniques that encourage unnecessary consumption, such as “buy three, pay for two” schemes [90].

Denmark’s Pharmacy Act and Health Act supplement this Nordic rigor by banning “loyalization” techniques that could tether patients to a pharmacy through commercial reward structures rather than therapeutic need [19].

Portugal’s Ordem dos Farmacêuticos dedicates an entire deontological chapter to “Publicidade e informação,” allowing the dissemination of professional activity in objective terms while prohibiting comparative or subjective content, misleading material, and patient identification [54]. The Netherlands follows a sobriety paradigm under KNMP standards and the Dutch Medicines Act, which discourages aggressive commercial advertising and frames the pharmacy as a medical service provider whose public communications must remain informative and professionally restrained [53].

#### 3.4.2. The Interprofessional Medical Relationship

European pharmaceutical law constructs strict barriers between prescribing and dispensing to safeguard patient autonomy and prevent the capture of therapeutic decisions by commercial complicity.

Bans on Collusion and Fee-Splitting: Romania’s Code of Ethics enforces a strict separation between the prescriber and the dispenser, prohibiting any material understanding that would limit the patient’s right to choose [32].

Germany’s Apothekengesetz codifies an absolute ban on fee-splitting and commission arrangements between physicians and pharmacists, treating the pharmacist as an independent health provider rather than a commercial extension of medical practice [69]. Poland’s Pharmaceutical Law criminalizes collusion and subjects the pharmacist’s verification duty to severe legal scrutiny [103]. Slovakia’s Professional Code explicitly forbids lucrative arrangements between doctors and pharmacists that would steer patients toward specific dispensaries [55]. Italy’s FOFI Code of Ethics requires that collaboration remain strictly clinical, disciplining any promotional or economic nexus with the medical profession [48].

- *Digital and Systemic Separation*: Estonia’s Medicinal Products Act structurally severs the doctor’s control over the dispensing point by routing all prescriptions through a national electronic system that allows the patient to collect medication at any pharmacy, thereby eliminating geographic or economic tethering [40].

Slovenia’s Code of Pharmaceutical Ethics prohibits economic incentives tied to patient steering and mandates that the pharmacist independently verify prescriptions, contacting the prescriber solely to correct errors or mitigate therapeutic risk [56]. Hungary’s Medicinal Products Act goes further by prohibiting the same person from simultaneously prescribing and dispensing to the same patient when a direct financial interest exists [105]. Denmark’s Pharmacy Act blocks any secondary activity, creating a conflict of interest in impartial dispensing, and forbids the simultaneous practice of medicine and pharmacy [19].

The Netherlands, Finland, and Sweden frame the physician-pharmacist relationship as a partnership within primary care teams, yet they preserve pharmacist independence through mandatory professional guidelines and digital protocols that prevent commercial influence [53,57,90]. Portugal’s Ordem dos Farmacêuticos integrates competition and deontology rules to promote interprofessional collaboration while banning any conduct that would subordinate therapeutic decisions to market incentives [54].

#### 3.4.3. Models of Inter-Pharmacy Competition

European pharmaceutical regulation bifurcates into two distinct models for managing inter-pharmacy competition: the non-commercial public-service model and the regulated market model.

- *The Non-Commercial Public-Service Model*. In Romania, the deontological code explicitly brands the attraction of patients through material advantages or false advertising as unfair competition, subjecting such conduct to disciplinary sanctions ranging from reprimand to practice prohibition [32]. This strict deontological position is set in a highly competitive market, where the economic and legislative factors, such as liberalization, have determined a rapid growth in the number of pharmacies in Romania in the last few decades [106].

Estonia characterizes the non-commercial approach by treating pharmaceutical activity as a health service rather than a market commodity: prices of reimbursed medicines are fixed nationally, generic substitution follows legal rather than commercial criteria, and pharmacies are barred from offering material incentives tied to prescribed medicines, forcing competition to revolve solely around the quality of professional counseling and service [40]. Slovenia similarly channels competition through accessibility, pharmaceutical counseling, supplementary services, and over-the-counter ranges, while prohibiting the use of prescription drug prices as competitive instruments and banning financial advantages linked to prescribed treatments [56].

- *The Regulated Market Model*: This model is exemplified by the Netherlands, Portugal, and Sweden, and tolerates competitive differentiation provided it remains subordinate to professional respect [36,54,57].

The Netherlands channels competition through health insurance contracts, removing prescription drug prices as a competitive variable and focusing rivalry on service quality and clinical outcomes [36]. Portugal integrates competition rules directly into its deontological code, outlawing unfair competition and misleading advertising while explicitly shifting the competitive focus away from market share and toward the dignity of the profession [54]. Sweden permits competition within a liberalized commercial framework, yet prohibits price-based rivalry on prescription medicines, instead differentiating pharmacies through extended hours, supplementary services, accessibility, and digitalization under strict authority control [57].

Hungary and Italy demonstrate how Continental systems use legal pricing walls to sterilize prescription-drug competition [48,105]. Hungary’s Medicinal Products Act enforces uniform national prices for prescription medicines, rendering promotional reductions illegal and redirecting competition toward pharmaceutical services and accessibility. [105] Italy’s FOFI Code mandates that competition remain loyal, banning aggressive advertising, the denigration of colleagues, and any unfair practice used to poach patients [48]. Poland adds a hybrid layer by partially regulating competition: while reimbursed drug prices are state fixed, pharmacies may differentiate through operating schedules and geographic coverage, though always within the boundaries of the Pharmaceutical Law [103].

#### 3.4.4. Professional Incompatibilities

The structural logic of professional incompatibility legislation across Europe reflects a fundamental concern: whenever a pharmacist’s economic interests intersect with therapeutic decisions, clinical judgment becomes vulnerable to distortion. Incompatibility legislation prevents clinical judgment from being distracted by intersecting economic interests. In light of these considerations, two distinct models exist with notable national variations: the absolute separation model and the commercial interest restriction model.

- *The Absolute Separation Model*: This model prohibits pharmacists from simultaneously practicing medicine, dentistry, or veterinary medicine.

Denmark enforces this with particular rigor: although a person may hold both pharmaceutical and medical degrees, simultaneous exercise of both professions is prohibited by law [19]. Germany reinforces this through the Fremdbesitzverbot, which prohibits non-pharmacist ownership of pharmacies entirely, preventing commercial investors from pressuring dispensing decisions [87].

- *The Commercial Interest Restriction Model*: This model targets vertical integration within the pharmaceutical supply chain.

The Netherlands prohibits any form of incentives or advantages offered by manufacturers or distributors that could influence the pharmacist’s professional decision, with acceptance of financial benefits from the pharmaceutical industry being strictly forbidden [53]. Austria maintains a comparable restriction model: rather than imposing a restriction on incompatibility between pharmacy and medical practice, it limits commercial control over pharmacies and regulates doctors dispensing under specific conditions [37]. Greece adopts parallel restrictions: legislation provides clear prohibitions on simultaneous involvement in wholesale distribution or in the manufacturing industry of medicines [46]. Denmark extends this logic by prohibiting pharmacists from holding interests in wholesale distribution or in the production of medicines, with any secondary activity that could create a conflict of interest in impartial dispensing being restricted by law [19]. Ireland applies a similar principle through the Pharmaceutical Society of Ireland (PSI) code; pharmacists must avoid prohibited financial relationships with healthcare professionals and manage professional interests that could compromise professional independence [21,22].

- *The Prescription-Dispensing Separation Rule*: This rule emerges as the fundamental principle across jurisdictions.

Italy establishes that professional incompatibility is intended to prevent a conflict of interest, such as incompatibility between participation in pharmacy operating companies, pharmaceutical manufacturing, or the scientific-information sector, and the exercise of the medical profession [48,107]. Hungary permits dual medical-pharmaceutical qualification but prohibits the professional from prescribing and simultaneously dispensing medicines to their own patients, with direct economic relationships between the medical act and the pharmaceutical act being expressly forbidden [71]. Slovakia prohibits lucrative co-practice between doctors and pharmacists in the provision of care and relationships that could enable unauthorized access to medicines [55].

- *Restrictions on Public Office and Concurrent Employment*: These restrictions appear in several frameworks.

Romania specifies that the profession is incompatible with simultaneous practice of medicine, with pharmacists in public office permitted to work outside hours only if this does not affect independence; medical conditions and criminal convictions also constitute grounds for incompatibility [104]. Cyprus identifies multiple restrictions: simultaneous practice in incompatible workplaces (public and private sectors), criminal convictions, inappropriate behavior, records, and medical unfitness [75].

**Figure 3:**
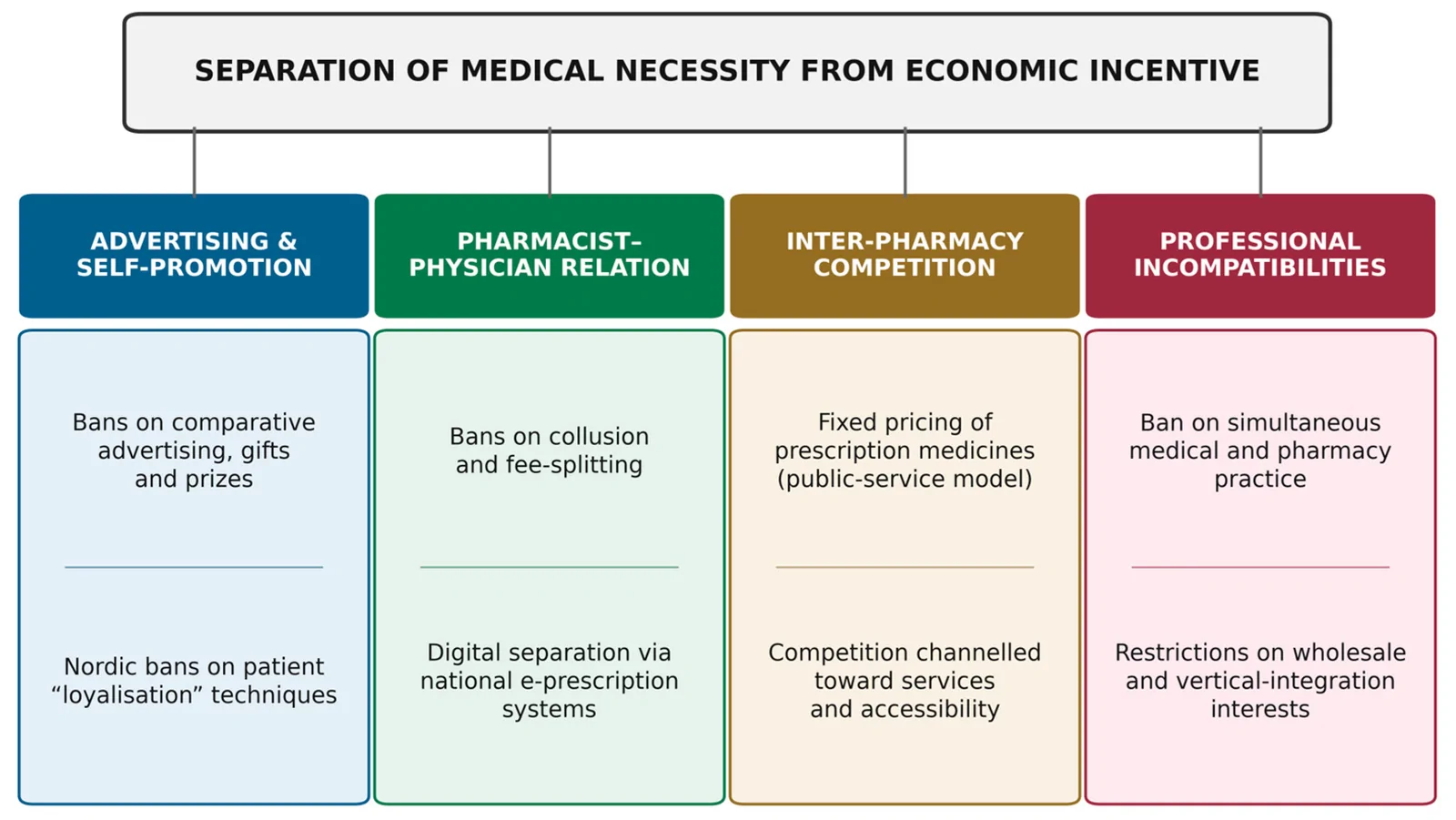
Four regulatory domains, deontological and legal protections safeguard a pharmacy practice from commercial pressure.

On another note, Finland addresses the conflict between therapeutic and commercial roles by restricting pharmacists from holding shares in manufacturing or wholesale distribution companies, guaranteeing neutral dispensing decisions, while also limiting concurrent practice of both medical and pharmaceutical professions to prevent profit generation from self-generated prescriptions [72]. The Commercial Environment of European Pharmacies is structured in four parts in Figure 3.

### 3.5. Contemporary Challenges: Telepharmacy, Digitalization, and Continuing Education

The modern pharmacist works at the intersection of rapid technological acceleration and ever-evolving clinical demands. Adapting to these contemporary challenges requires not only a solid regulatory framework for distance selling and digital health tools but also structured models of continuing education to maintain professional competence.

#### 3.5.1. The Telepharmacy Landscape and Digital Paradigms

Telepharmacy is defined as the provision of pharmaceutical care through digital technologies. Patients increasingly seek professional advice on their treatment, side effects, and treatment adherence. Telepharmacy offers pharmacists the ability to deliver remote consultation, prescription verification, or adherence support via secure online platforms.

The European landscape of telepharmacy and online medicines is defined by two distinct regulatory models: balancing patient accessibility with professional oversight, for example, the liberalized digital paradigm and the restrictive OTC-limited paradigm.

- *The Liberalized Digital Paradigm.* This model treats online prescription medicine dispensing as a standard care modality fully integrated into the national health system, and is frequently found in Nordic and Baltic infrastructures.

Denmark operates the most advanced version of this framework, where 99% of prescriptions are electronic, and the national Apoteket application enables real-time stock visibility, online ordering, and call counseling before home delivery, with the regulatory architecture governing the entire process sourced from the Danish Medicines Agency and the Pharmacy Act [19].

Finland achieves a similar architecture, achieving 99% electronic prescription coverage, treating online pharmacies exclusively as a digital extension of physical establishments, and mandating telephone counseling before parcel dispatch [72]. Sweden mirrors this pattern through near-universal e-prescription deployment, with OTC medicine sales regulated through the EU common logo [34]. Estonia integrates the pharmaceutical act into the mandatory national digital health system, requiring electronic prescription verification for all online dispensing, automatic data logging for traceability, and pharmacist access to complete medication history through patient electronic identification [40].

Norway functions as a pan-European laboratory for digital pharmacy, authorizing online prescription medicine sales as standard practice with mandatory BankID identification and secure home delivery [108]. Also, they have implemented the platform e-Prescription II, which is used as a crucial service for hospitals and supports multi-dose dispensing [109].

- *The Restrictive OTC-Limited Paradigm.* The second model constitutes the majority position across the continent. Italian legislation explicitly prohibits online sales of prescription medicines, because they can only be sold in pharmacies, while authorizing over-the-counter products under the following conditions provided by Article 112-quarter of the Drugs Code. When selling products other than medications, pharmacists are required to act in accordance with their role in the healthcare system, in the interest of public health and the professional image of pharmacists [48,110].

Austria restricts online commerce to non-prescription medicines exclusively, requires pharmacies to hold explicit distance selling authorization, obliges remote patient counseling upon request, and regulates the entire activity through the Pharmacy Law and Distance Selling Procedure [111]. Ireland confines online medicine supply to OTC products, mandates registration with the Pharmaceutical Society of Ireland (PSI), requires display of the EU common logo on every web page offering medicine, and specific package size safety measures [112]. Malta permits online pharmacy activity only where the physical establishment holds a license, restricting digital activity to over-the-counter medicines, drawing the regulatory framework from medicines legislation and EU norms on online medicine sales [52]. Spain operates on a similar OTC-limited basis, with mandatory EU logo linkage and video consultation in the pilot phase [113].

Several jurisdictions impose additional structural constraints and Absolute Prohibitions beyond product category restrictions. Belgium requires that any online pharmacy site functions exclusively as a digital extension of a licensed physical pharmacy, prohibiting any other products or services beyond what is authorized in the physical establishment [38]. Cyprus maintains an absolute prohibition on online pharmacies and online medicine sales under the Pharmacy and Poisons Law, representing the most restrictive position documented, with future EU common logo registration the sole pathway to any digital pharmacy activity [75]. Portugal authorizes distance selling of over-the-counter medicines, subject to mandatory communication to INFARMED and display of the EU common logo [114].

The convergent ethical imperative across all regulated models is pharmacist-led counseling in the digital environment. Mandatory video or telephone counselling before dispensing, traceability of the pharmaceutical act through electronic prescription systems, and the EU common logo as the primary legitimacy marker constitute the shared regulatory vocabulary, irrespective of the degree of digital integration. The fundamental tension remains: the liberalized Nordic model prioritizes accessibility and system integration, while the majority of European jurisdictions prioritize the physical pharmacy presence as the guarantor of professional oversight in an online transaction.

In the context of Europe’s evolving health landscape, telepharmacy is considered a strategic response to systemic challenges, and these services do not replace traditional brick-and-mortar pharmacy’s services; they just complete them. At the same time, telepharmacy acts as a connection between underserved communities and healthcare professionals, particularly in rural and suburban areas where unequal distribution of healthcare professionals is increasingly common. Access to telepharmacy services remains highly fragmented across Member States, leading to unequal access to healthcare services for European citizens. Some countries, such as Sweden, Germany, and the Netherlands, have successfully integrated telepharmacy into their national health systems [115].

In the case of countries with low population density, such as Sweden or the United Kingdom (Scotland), telepharmacy is structurally integrated as an essential healthcare service, especially for rural older adults to improve their access to clinical pharmacy, and is legally accepted [116]. In the United Kingdom, telepharmacy is not regulated as a separate category, but rather integrated under general healthcare and pharmacy legislation by the General Pharmaceutical Council [62]. A study conducted by Jackie I. (2017) highlighted telepharmacy in rural Scotland (UK), where the technology enables medical services to be delivered to rural populations. This concept is based on the community pharmacy services, including advice, sale of OTC products, and dispensing of prescriptions by tele-technology (The Telepharmacy Robotic Supply Services) [117].

In France, telepharmacy is authorized and includes two forms of remote patient care that use videotransmission: teleconsultation and tele-care (téléconsultation et le télésoin), and remote pharmaceutical monitoring is accepted, especially for supporting aging populations in depopulated rural areas [118]. On the other hand, in Romania, telepharmacy is not fully regulated, as Law 95/2006 reveals that there are some limitations and shortcomings caused mainly by the general and undifferentiated nature of primary legislation. Current legislation recognized telemedicines as a means of providing medical services, but does not establish clear criteria for its differentiated applications [104].

#### 3.5.2. Integrating Digital Transformation and Ethical Artificial Intelligence

As highlighted by recent literature, this digital expansion goes beyond simple e-commerce.

A study conducted by Wahl et al. (2024) on the digital transformation of pharmacy services in Germany demonstrates that advanced technologies and artificial intelligence are fundamentally restructuring how care is delivered, expanding accessibility but complicating regulatory compliance and data security [119]. As future trends and opportunities in digital pharmacies are highlighted, a few innovations, such as machine learning, artificial intelligence, or blockchain. Artificial intelligence can be applied to create individualized therapy for patients by utilizing personalized patient data and machine learning algorithms that could reveal general patterns in medication usage. However, the introduction of AI-based decision-support tools introduces novel ethical and legal dilemmas.

As argued by Cartolovni et al. (2022), these software tools directly impact pharmacist accountability and the traditional pharmacist-patient relationship, reinforcing the urgent need for “Ethics by Design” principles to be integrated into pharmaceutical digital platforms to ensure algorithms preserve patient safety and professional independence [120].

At the EU level, two legislative instruments frame the field. Under the Artificial Intelligence Act, an AI system that is itself a medical device or a safety component of a medical device, and that is subject to third-party conformity assessment under medical device legislation, is classified as high risk under Article 6(1) [121]. Software that supports clinical decision-making used in pharmacy practice falls within this category. As a result, providers of such software or systems must design them so they can be effectively overseen by natural persons capable of interpreting the output, remaining aware of automation bias, and able to disregard, override, or reverse it, according to Article 14. The European Health Data Space Regulation sets an EU framework for the use of primary and secondary electronic health data, and electronic prescription and dispensing of medicines are among the priority categories of data to be exchanged across borders. The deontological codes examined in this paper do not address the obligations introduced by these two instruments.

As clinical practice increasingly interacts with artificial intelligence, European pharmaceutical regulatory frameworks urgently need to define the ethical parameters of algorithmic care. Currently, there is a significant institutional divergence: while some jurisdictions are actively incorporating artificial intelligence as a certified medical technology, others depend on professional bodies to issue protective guidelines that emphasize the irreplaceable nature of human judgment. In the next paragraphs, several issues related to certain countries will be addressed.

Regarding Italy, the Code of Ethics of the FOFI, the current written version of 2018, does not include explicit references to artificial intelligence and considers it just a support tool and not an idea of substitution for the pharmacist’s ethical and critical judgment. All aspects related to the use of artificial intelligence in this sector follow specific indications [48].

A recent publication from June 2026 reveals the collaboration between the Italian Medicine Agency (L’Agenzia Italiana del Farmaco—AIFA) and the Federation of Italian Pharmacists’ Orders (Federazione degli Ordini dei Farmacisti Italiani—FOFI) with the aim of developing a new digital solution to support pharmaceutical assistance. FOFI will make available a service based on artificial intelligence that integrates various information, such as regulatory information on medicines, with professional support tools designed for pharmacists. This implementation aims to support the pharmacist in the daily exercise of the profession, and particular attention will be paid to activities to monitor chronic pathologies and promote therapeutic adherence [122].

In Germany, there has been an impressive push towards the digitalization of pharmacies and the integration of artificial intelligence into the healthcare system. According to the Digital Health Care Act (DVG—Digitale-Versorgung-Gesetz), doctors can prescribe health apps for patients, such as apps for monitoring chronic diseases and apps for tracking their blood sugar levels. After the apps are approved by the Federal Institute for Drugs and Medical Devices and checked for security, quality, and functionality, it will be provisionally reimbursed by statutory health insurance for one year [123].

In the United Kingdom, the Medicines and Healthcare products Regulatory Agency (MHRA) regulates artificial intelligence as a Medical Device. In June 2023, MHRA announced that artificial intelligence plays a key role in healthcare and social assistance [124]. In addition, the National Institute for Health and Care Excellence (NICE) states that artificial intelligence represents a significant opportunity to better deliver its core mission, and it can help improve health outcomes and system productivity, drive innovation within the life sciences and health technology sectors [125].

In light of the above, there is a major discrepancy between technical health legislation and pharmacist’s code of ethics. Some countries, such as Germany and the United Kingdom, have begun to certify artificial intelligence-based clinical applications, but pharmacist’s code of ethics has not been updated with this topic. The worst part of this subject is the fact that healthcare specialists have no guidance on how to interact with these tools or to assume legal and moral responsibility when using AI support.

#### 3.5.3. Continuing Pharmaceutical Education (CPE) Models

Across Europe, two distinct models govern pharmacist continuing education, maintaining lifelong professional competence. The two fundamental models are the mandatory credit system model and the ethical/voluntary model.

- *The Mandatory Credit System Model*: This model ties professional development directly to license renewal through quantified requirements, and includes several countries, like Slovenia, Ireland, the United Kingdom, Cyprus, and Romania.

Slovenia, under the Pharmacy Practice Act, requires pharmacists to accumulate continuing education points accredited by the Chamber of Pharmacists, where non-compliance potentially leads to suspension of practice or restriction of services funded by health insurance [56,89]. Ireland operates an annual mandatory continuing professional development system monitored by the PSI, where pharmacists must participate in accredited courses, conferences, online learning, clinical audits, and reflective practice, with failure potentially affecting the right to practice [73]. The United Kingdom employs a structured annual revalidation under GPhC standards [25]. Cyprus requires pharmacists to participate in continuous professional education; however, there is no mention of the renewal of the pharmacist’s license. Romania requires 40 EFC credits annually, representing elevated standards [75,126].

- *The Ethical/Voluntary Model*: This approach frames continuing education as an intrinsic professional duty without direct point-based linkage to license renewal.

In Sweden, it operates without a rigid point system, but employers and regulatory authorities continuously monitor professional competence as an implicit quality standard [127]. Finland lacks a rigid points system, but employers face a legal obligation to ensure staff professional development and ensure enough participation in continuing education. [72,90,128]. Estonia imposes continuing education as a legal condition for practice, yet without a mandatory credit system managed by a professional chamber, placing responsibility for competence maintenance on the individual practitioner [79]. Malta does not condition license renewal on continuing education, though local academic work contributes to competence maintenance [76].

Institutional responsibility varies. In some jurisdictions where accreditation powers are vested in professional bodies, they are also responsible for defining the requirements for continuing development, organizing or approving learning activities and maintaining accreditation, as we have in Slovenia, Ireland or Romania [21,89,104]. In Great Britain, the GPhC is responsible for the verification function following a yearly revalidation against professional standards [129].

## 4. Discussion

### 4.1. Ethical Convergence Versus Legal Divergence in the European Space

The comparative analysis of the 30 states’ deontological codes revealed an ethical core centered on the patient; however, their legal architecture and enforcement powers differ significantly. Common ethical principles were also evident in relation to grounds for refusing to dispense medicines, conscientious objection, professional secrecy and confidentiality, incompatibilities with other professions in the medical field, and restrictions on marketing policy. Nonetheless, identifying elements of convergence among these codes is not equivalent to European harmonization. 

The study found three regulatory models regarding the status of the codes: some states introduced norms into the health legislation concerning ethical standards for pharmacists; other states transferred the “normative power” to professional bodies, which have elaborated their own deontological codes; and a few states have resorted to the “soft” mechanism. 

This division is in line with the recent study by Raimundo and Cavaco (2024), which found that the core values of honesty, integrity and professional autonomy underpin pharmacy practice in culturally diverse European countries [6]. None of the national codes fully aligns with the proposed FIP framework, resulting in major structural inconsistencies. Nevertheless, our study suggests that these inconsistencies are largely legal and operational. The translation of ethical obligations into either mandatory primary legislation (e.g., Estonia) or quasi-legal corporatist codes (e.g., Bulgaria, Poland or Romania) directly affects the disciplinary power and the practical application of the deontological framework. 

The divergence in enforcement lies in the distinction between “hard law” and “soft law”. As the analysis has shown, some jurisdictions have made ethical norms binding, with the consequence that legal liability can arise. Other states have limited themselves to incorporating certain guiding principles of pharmacists’ professional conduct into their health legislation, without imperative normative force. The consequences are also evident in potential liability if these norms are breached, and here the divergences are significant (ranging from the finding of a disciplinary offense to criminal liability).

### 4.2. Professional Independence: Protected or Vulnerable?

The analysis of deontological codes and regulations of various states revealed that the professional independence of pharmacists remains open to question, irrespective of how it is regulated, and therefore, the expansion of pharmacists’ responsibilities is directly proportional to certain commercial pressures, while patients’ needs come second [130].

In this context, a legislative inconsistency arises from the lack of a common approach at the European level. Thus, while some states have explicit provisions prohibiting unfair competition or other forms of interference with pharmaceutical practice, others merely set general standards within broader healthcare legislation.

On the other hand, the theoretical solution adopted by the legislator—the express nomination of professional independence—does not automatically provide genuine protection. As long as some systems allow pharmacists’ salaries to be contingent on economic performance linked to sales of particular products or the achievement of a commercial target, professional independence is reduced to a merely declared objective [131]. This is consistent with the observation that the ethical codes support professional autonomy, yet they do not address the dual loyalties and commercial pressures that often undermine it in practice [6].

It has been further shown in the literature that in recent years, business interests and commercial pressures have intensified, and the deontological codes and regulations in their current form no longer serve the purpose for which they were planned and adopted, including the impact of digitalisation on pharmacy practice and the development of pharmacy chains [14,132].

Commercial pressure is not limited to pharmacists working in corporate structures. In cases where ownership is limited to pharmacists, the conflict is not removed but internalized: the owner, whose income depends on turnover and margin, has exactly the same incentive to increase sales, exercised on themselves rather than by an employer. The two configurations differ in the source of pressure, not in its existence. In markets that allow 3rd-party ownership, the chain management layers may add an external level—sales targets, performance-linked bonuses and centrally determined commercial policy—that an employed pharmacist has difficulty refusing. Deontological codes address the pharmacist as an individual moral agent and are therefore ill-equipped for any of these configurations: they prohibit subordinating professional judgment to commercial interest without regulating the structures that generate that interest.

The solution that emerged from the legal texts consulted—an express statement that the pharmacist enjoys professional independence, that commercial interests may not interfere with it and that incompatibilities are named—remains theoretical without enforcement. Effectiveness requires intervention in three places: the sanctions applied when the Code is breached, the determination of remuneration and pharmacy ownership.

A reference point in this context is Norway, which ensures the protection of pharmacists’ independence through the transparency of professional practice resulting from digitalisation and the application of sanctions when needed [20,109]. At the opposite pole stands Romania, where despite express regulation, certain vulnerabilities persist. The market liberalization that drove the rapid growth in the number of pharmacies intensified commercial pressure on the dispensing [9,133]. Consequently, the mere existence of a normative framework without concrete mechanisms to protect independence remains form without substance.

### 4.3. The Pharmacist as a Public-Health Actor: Recognized or Ignored?

In the current context of Europe, marked by an aging population and health budgets choked by rising costs, the properly regulated pharmacist-led pharmacy becomes a strategic solution for the sustainability of the healthcare system. Regulation and ethics shape the pharmacist’s activities in favor of public health along several lines [134].

The pharmacist’s role has evolved under regulations that expand their role in prevention and primary care. Provided adequate training and equipment are available, the pharmacy becomes an accessible setting for screening and monitoring: pharmacists perform rapid tests (glycemia, cholesterol, blood-pressure monitoring), contributing to the early detection of chronic diseases such as diabetes and hypertension. The legal recognition of these activities as professional services varies across jurisdictions in scope, as in some systems they are formalized as a dedicated service, while in others they are treated as a monitoring function. [135,136]. While Italy implemented screening pharmacy services (hepatitis-C), in Austria such testing is a monitoring function, these being the two poles (Section 3) [37,99,135,136,137].

The same logic applies to vaccination campaigns, with the regulation of influenza or COVID-19 vaccination in pharmacies already implemented in many European countries. Depending on the healthcare system, these services are either recognized and billed separately in countries that consider intervention as a pharmaceutical service or included in the product price through dispensing activities. For example, Belgium implemented pharmacist-administered vaccination as a reimbursed pharmacy service. However, the adoption of pharmacist-administered vaccination is not yet widespread [137,138].

Secondly, new frameworks in healthcare systems emphasize the role of pharmacists in polypharmacy, especially for older patients who are both important consumers of medicines as well as patients with comorbidities. Guided by ethical principles and regulated by clear protocols, the pharmacist can review an elderly patient’s therapeutic regimen to identify dangerous drug interactions, adverse reactions, or treatment duplication [139,140]. This type of review is included in the pharmaceutical services formulary in regions with a service model. In other countries, therapy verification is considered part of dispensing, with its value arising from the product price [141]. Germany or remunerate activities such as medication review, whereas Estonia or Hungary consider therapy verification part of a broader dispensing concept and incorporate its value into the price of the medicine dispensed. Furthermore, EU law reinforces the core principle by acknowledging pharmacies as healthcare facilities instead of typical commercial businesses [142]. This recognition justifies restrictions aimed at safeguarding professional independence and clinical judgment from commercial influences.

Clinical pharmacy competencies enable pharmacists to implement clinical strategies that contribute to cost optimization. Regulations often oblige or incentivize pharmacists to propose generic and biosimilar alternatives, reducing patients’ out-of-pocket expenses. Regulated counseling that improves adherence helps patients understand how and why to follow their treatment, averting the complications and expensive hospitalizations associated with non-adherence. Pharmaceutical services linked to adherence and generic substitution have been included in standard contractual obligations in several countries or are under structured evaluation in other countries [143,144].

Lastly, patient safety is linked with responsible use of self-medication, and the pharmacist is assigned by regulation a role in this field. In an era of online disinformation, the pharmacist ensures that the information provided in the pharmacy rests on scientific evidence. The restriction of certain active substances to licensed personnel under strict rules prevents the misuse of antibiotics, strong analgesics, and other medicines, while strict supply-chain rules — such as the European Falsified Medicines Directive—ensure that patients receive authentic products [145].

The pattern we see across both criteria (Section 3.3.3 and Section 3.3.4) is that the public health role of the pharmacist originates from general health legislation and reimbursement systems rather than from deontological codes, a pattern well documented [146]. An exception documented by our data is Italy, where continuity of pharmaceutical care is governed by the deontological code itself in conjunction with the national healthcare framework as presented in Section 3.3.3.

### 4.4. Digital Gap and AI

The deontological problems that may arise in pharmacy practice in the context of digitalization are not fundamentally different than those present currently in the practice. In some circumstances, they may amplify existing vulnerabilities. In this regard, the thesis has been advanced that the traditional codes are not adequate to the requirements of online care so that an adaptation of their content is necessary.

It is important to emphasize that telepharmacy, or the delivery of pharmacy services in the online environment, should not be conceived as a faster and more efficient alternative. Professional and pharmacy industry bodies argue that it must be seen to complement, not replace, community pharmacy and be framed as an extension of professional activity that must have the same guarantees of security, responsibility, and protection of confidentiality on the professional side [115]. 

A comparison of different normative acts that regulate the ethical conduct of pharmacists has shown greater openness to dispensing OTC medicines through digital applications, and convergence in the restrictions applied to dispensing prescription-only medicines. This is perfectly justified. 

On the other hand, it is possible to identify various vulnerabilities inherent in pharmacy activity in the digital environment, for which the legislator will have to find solutions. A first debatable aspect is the way in which digital platforms may use various algorithms for marketing strategies (e.g., scoring and upselling) [147]. This problem has also been identified by other authors, who pointed to the lack of a normative framework in this regard [14]. There are states with a high degree of digitalization that manage such risks well, as their platforms are designed to ensure transparency of professional activity and are integrated into the public health system. However, applications that provide online pharmacy or pharmaceutical advice are not yet linked with public health platforms.

The regulatory gap is evident in the field of artificial intelligence. AI-based tools are already used in pharmacy practice, ranging from clinical decision support to large language models [148,149]. The gap has become deeper since the adoption of the Artificial Intelligence Act and the European Health Data Space Regulation [121,150]. While the first imposes an important duty of human oversight for deploying high-risk clinical AI, the second opens the possibility of exchanging data across borders. Both assume the existence of a professional who applies independent judgment to algorithmic output and the data produced. This is precisely the figure the deontological codes describe and the situation for which they provide no guidance. The ethical challenges relate to accountability for AI-based decisions, transparency, liability attribution, and traceability [120]. Yet the deontological codes analyzed lack norms on these instruments: professional responsibility formally remains with the pharmacist, but there is no deontological guidance on how it is to be exercised when recommendations come from algorithms or AI-based tools. Therefore, while telepharmacy and online pharmacy have started to receive at least fragmentary regulatory solutions, the use of AI in pharmacy practice remains largely unregulated.

Also, elements that can generate uncertainty include those related to the person responsible for the digital platform, for which the deontological code should include explicit rules, particularly in the fields of confidentiality protection and traceability.

### 4.5. Implications for European Coordination

Any movement toward the harmonization of European deontological codes must be within the legislative competence of the Union, the point of reference being Article 5(2) of the Treaty on European Union, according to which the Union acts only within the limits of the competences conferred on it by the Treaties [151]. 

The provisions of the Treaty on the Functioning of the European Union (TFEU) imply that the field of public health—and, implicitly, pharmacy practice—is not among the Union’s exclusive competences, so that the Union cannot fully replace national competences, but only implement certain mechanisms in support of the Member States. Furthermore, article 168(7) of the treaty specifically says: “*Union action shall respect the responsibilities of the Member States for the definition of their health policy and for the organisation and delivery of health services and medical care*.” [152].

Based on the above, the European Union cannot directly harmonize national laws into a single deontological code but can indirectly promote this goal by establishing a set of mandatory minimum standards. Member States maintain sovereignty over how these standards are implemented locally—a similar approach taken by Directive 2005/36/EC on recognizing professional qualifications.

These provisions are in line with the case-law of the Court of Justice of the European Union. In this respect, the judgment in Joint Cases C-171/07 and C-172/07 (Apothekerkammer des Saarlandes and Others (C-171/07) and Helga Neumann-Seiwert (C-172/07) v Saarland and Ministerium für Justiz, Gesundheit und Soziales) is of particular importance [12] because the Court was asked whether Union law precludes national provisions that prohibit persons other than pharmacists from owning and operating a pharmacy. The Court held that the freedom of establishment and the free movement of capital do not preclude such national rules, provided that the objective pursued is legitimate. The judgment in Joint Cases C-159/12 to C161/12 (Alessandra Venturini v ASL Varese and Others Maria Rosa Gramegna v ASL Lodi and Others and Anna Muzzio v ASL Pavia and Others) is also illustrative as the Court reaffirmed the discretion of the Member States in the organization of their health systems [153].

However, the European Union is not entirely deprived of a margin of cation in this field: Directive 2005/36/EC was adopted as an instrument to harmonize professional qualifications, although not the ethical standards. Furthermore, Directive 2006/123/EC on services in the internal market refers to the adoption of professional codes of conduct [154]. Two conclusions could be drawn—on the one hand, it assumes an asymmetry in the deontological codes at the national level and on the other hand, it does not require the development of a single normative framework with binding legal force.

Another element to consider is the principle of subsidiarity (Article 5(3) TEU), under which the Union shall act only if the objectives of the proposed action cannot be sufficiently achieved by Member States [151,155]. From this perspective, the question is whether harmonizing deontological codes would be necessary to that extent, or whether the way in which they have been developed at the domestic level is appropriate to the purpose for which they were adopted. 

In this context, an issue that may be raised is related to the difference between statute law and case law. As can be seen from the analysis conducted, most countries have regulated the ethical standards of the pharmacy profession through various written instruments (laws, codes of ethics, etc.). However, the manner in which these are interpreted and applied to specific situations remains the purview of the courts. For example, in France, the Conseil d’État has clarified the content of certain ethical obligations by referring to the specific facts of the case under review [156]. Furthermore, the way in which case law can influence statutory law is also reflected in the sanctions applicable in the event of a violation of legal norms. We state this because judicial practice can either strengthen or, conversely, diminish the effectiveness of certain legal provisions.

It should be noted, however, that in civil law systems, case law does not constitute a source of law; a court decision is binding only on the parties to the dispute. Nevertheless, there are certain mechanisms for standardizing judicial practice (for example, in Romania, an appeal in the interest of the law may be filed when lower courts have issued differing judgments on the same legal issue, and the ruling thus issued by the High Court of Cassation and Justice becomes binding). Even at the European Union level, there is the mechanism of preliminary rulings (Art. 267 TFEU) for the “uniform interpretation and application of Union law” [157]. Furthermore, through preliminary rulings, the CJEU clarifies the meaning, scope, or content of certain legal rules [158].

Essentially, the interpretation of the content of ethical standards (regarding what constitutes a disciplinary violation, how sanctions are applied and their proportionality, their compatibility with European law, etc.) remains within the jurisdiction of the courts, which may expand or limit the scope of the ethical rules depending on the specific circumstances. On this point, the answer must be qualified. The analysis of deontological codes shows a certain convergence regarding values, focus on the patient, professional competences, honesty, reasons for refusing to dispense medicine, respect for confidentiality, and certain norms on advertising. On the other hand, telepharmacy and the use of AI in pharmacy practice require an appropriate legislative response. A common minimum deontological standard covering professional independence, confidentiality, errors, and digital impact on pharmacy practice could be promoted by the PGEU for European coordination.

These pressures are not specific to Europe. The question of payment for community pharmacy services is a recognized global problem, and the use of commercial sales tactics that influence dispensing has been reported in markets far beyond Europe [141,147]. International comparisons of codes of ethics show similarities, particularly on issues such as conscientious objection, which are discussed in similar terms within and outside Europe [16]. The main difference for Europe is not the nature of these challenges but the density and complexity of the tools developed to address them. The reference point globally remains the standards set by FIP, either on its own or in partnership with the World Health Organization [7,8]. These are not binding norms but act as benchmarks for the development and evaluation of national codes. The aim in Europe is to ensure that its tools remain in line with practice, not to replace them with one single, unified code.

### 4.6. Limitations

A limitation of this study is that documents available only in national languages were machine translated; however, all extracted data were verified against the cited primary sources by two of the authors, and nuances of national legal terminology may have been lost in translation.

The study is based on document analysis of legal texts and thus covers the formal content of deontological frameworks, not their actual enforcement or the daily practice of professional bodies.

The regulatory landscape for digitalization and AI is rapidly evolving. Our findings reflect the state of documents collected between April and June 2026.

The comparative analysis is qualitative. No quantitative coding was performed per jurisdiction, and cross-country statements should be read as descriptive patterns rather than counts.

## 5. Conclusions

This study examines whether the deontological codes and frameworks of 30 European countries ensure enough support for contemporary pharmacists’ roles, focusing on professional independence. It finds a shared ethical core—patient priority, confidentiality, honesty- while legal differences based on legislation type affect enforceability. Professional independence is consistently recognized across the frameworks analyzed, and yet key factors like remuneration, pharmacy ownership model or incompatibilities fall outside strict regulations in many cases or remain merely declared objectives.

The expanding public health roles of pharmacists, such as screening, vaccination, medication review and adherence support, are largely driven by health legislation and reimbursement systems, rather than deontological codes. In the digital age, this gap is getting bigger: telepharmacy is subject to fragmented regulation, while the use of AI is largely untouched by deontological codes. 

The findings do not support supranational harmonization beyond the EU’s competences and subsidiarity. Instead, they promote a common minimum deontological standard that includes professional independence, confidentiality, errors, and digital practice. Professional bodies with PGEU are advocating for the profession’s interests at the European level. Therefore, future research should include per-jurisdiction coding of the code texts and empirical studies on the level of enforcement.

## Figures and Tables

**Table 1:** The 14 comparison criteria are grouped into five thematic clusters.

Thematic Cluster	No.	Criterion	Analytical Focus
T1 Legal architecture	C1	Legal status of the code	Binding normative act vs. good-practice guide; legal validity, publication, enforceability
T2 Professional independence and its safeguards	C2	Professional independence	Priority of patient health over commercial interest; limits set by the code
	C3	Refusal/conscientious objection	Right to refuse dispensing on personal, moral, or religious grounds; permitted conditions
	C12	Salary regulation *	Whether pharmacists’ remuneration is specifically regulated
T3 Pharmacist–patient relationship	C4	Professional confidentiality	Scope, limits, and exceptions of confidentiality
	C8	Professional error	Ethical handling of error, from minor mistakes to malpractice; duties and consequences
	C10	Continuity of care	Obligation to provide assistance in critical or emergency situations (e.g., on-call duty)
	C13	Pharmaceutical services	Which professional services are regulated, and who funds them
T4 Professional and commercial environment	C5	Advertising and image	Restrictions on marketing and self-promotion
	C6	Relationship with physicians	Prohibition of collusion over prescriptions
	C7	Competition/other pharmacies	Relationship with other pharmacists and pharmacies; fair vs. unfair means of attracting patients
	C14	Incompatibilities/conflicts of interest	Situations limiting practice: incompatibility and unfitness
T5 Contemporary challenges	C9	Telepharmacy/digitalization	Ethical regulation of distance dispensing: what is permitted online
	C11	Continuing education (CPE)	Linking ethics to technical competence, professional obligations

(*) Salary regulation is not in itself a deontological norm; it is included as a factor of professional vulnerability bearing on independence. The thematic clusters were applied after the extraction and did not structure the data collection phase.

**Table 2:** Distribution of deontological regulatory models in Europe.

Country	Model	Deontological Instrument (Original + EN)	Issuing Authority	Legal Force and Sanctions	Ref.
Austria	M2	Berufsordnung (Professional Code of Conduct)	Österreichische Apothekerkammer (Austrian Chamber of Pharmacists)	Binding + disciplinary	[37]
Belgium	M2	Code de déontologie pharmaceutique (Code of Pharmaceutical Deontology)	Conseil national de l’Ordre des pharmaciens	Binding + disciplinary	[38]
Bulgaria	M2	Koдeкc зa пpoφecиoнaлнa eтикa нa мaгиcтъp-φapмaцeвтa (Code of Professional Ethics of the Master Pharmacist)	Бългapcки φapмaцeвтичeн cъюз (Bulgarian Pharmaceutical Union, BPU)	Binding + disciplinary, administrative fines	[26]
Croatia	M2	Kodeks etike i deontologije farmaceuta (Code of Ethics and Deontology of Pharmacists)	Hrvatska ljekarnička komora (Croatian Chamber of Pharmacists)	Binding + disciplinary	[29]
Cyprus	M2	O περί Φαρμακοποιών (Σύλλογοι, Πειθαρχία και Ταμείον Συντάξεων) Νόμος (The Pharmacists (Associations, Discipline and Pensions Fund) Law)	Παγκύπριος Φαρμακευτικός Σύλλογος (Cyprus Pharmaceutical Association)	Binding + disciplinary	[39]
Czech Republic	M2	Etický kodex farmaceuta (Code of Ethics of Pharmacists)	Česká lékarnická komora (Czech Chamber of Pharmacists)	Binding + disciplinary	[28]
Denmark	M1	Bekendtgørelse af lov om apoteksvirksomhed (Consolidated Danish Pharmacy Act, Act No. 703/2023)	State (Lægemiddelstyrelsen/Danish Medicines Agency)	Legislation binding; no standalone ethics code exists	[19]
Estonia	M1	Ravimiseadus (Medicinal Products Act) + Tervishoiuteenuste korraldamise seadus (Health Services Organization Act)	Legislator (Riigikogu)	Binding + administrative/penal	[40,41]
Finland	M3	Farmasian ammattilaisen eettiset ohjeet (Ethical Instructions for Pharmacy Professionals) + Apteekkitoiminnan eettiset ohjeet (Ethical Instructions for Pharmacy Operations)	Suomen Farmasialiitto + Suomen Apteekkariliitto (professional associations)	Voluntary; guidance	[42,43]
France	M2	Code de Déontologie des Pharmaciens (Articles R4235-1 to R4235-64, Code de la santé publique)	Ordre National des Pharmaciens; approved by the Minister of Health	Binding + disciplinary	[44]
Germany	M2	Berufsordnung (Professional Code of Conduct of the State Chambers of Pharmacists) varies by Land	Landesapothekerkammern (Regional Chambers of Pharmacists)	Binding + disciplinary	[45]
Greece	M2	Κώδικας Επαγγελματικής Φαρμακευτικής Hθικής (Code of Pharmaceutical Ethics)	Presidential Decree 340/1993 (Ministry of Health)	Binding + disciplinary	[46]
Hungary	M2	A Magyar Gyógyszerészi Kamara Etikai Kódexe (Code of Ethics of the Hungarian Chamber of Pharmacists)	Magyar Gyógyszerészi Kamara (Hungarian Chamber of Pharmacists) +Legislator	Binding + disciplinary	[47]
Ireland	M1	Code of Conduct: Professional Principles, Standards and Ethics for Pharmacists	Pharmaceutical Society of Ireland (PSI) (statutory body under Pharmacy Act 2007)	Binding + disciplinary	[21]
Italy	M2	Codice Deontologico del Farmacista (Code of Ethics of the Pharmacist, 2018)	Federazione Ordini Farmacisti Italiani (FOFI)	Binding + disciplinary	[48]
Latvia	M3	Ētikas kodekss (Pharmacist Ethics Code)	Latvijas Farmaceitu biedrība (Pharmacists’ Society of Latvia)	Binding + disciplinary	[49]
Lithuania	M3	Farmacijos darbuotojų etikos kodeksas (Code of Ethics of Pharmacy Workers)	Lietuvos farmacijos draugija (Lithuanian Pharmaceutical Association)	Voluntary; guidance	[50]
Luxembourg	M1	Code de déontologie des pharmaciens (Code of Deontology of Pharmacists)	Collège médical; approved by Ministerial Order (Arrêté ministériel du 11 juillet 2011)	Binding + disciplinary	[51]
Malta	M1	Code of Ethics for the Pharmaceutical Profession	Pharmacy Council of Malta (Kamra Tal-Ispiżjara)	Binding + disciplinary	[52]
Netherlands	M3	Code van de beroepsgroep (Code of the professional group/Code van apothekers)	KNMP (Royal Dutch Pharmacists Association)	Voluntary; adopted as veldnormen	[53]
Norway	M1	Lov om apotek (Apotekloven/Pharmacy Act, 2000)	State (legislator)	Binding; outcome-based standards	[20]
Poland	M2	Kodeks Etyki Aptekarza Rzeczypospolitej Polskiej (Code of Ethics of the Pharmacist of the Republic of Poland)	Naczelna Izba Aptekarska (Supreme Chamber of Pharmacists)	Binding + disciplinary, criminal	[33]
Portugal	M2	Código Deontológico da Ordem dos Farmacêuticos (Deontological Code of the Order of Pharmacists)	Ordem dos Farmacêuticos	Binding + disciplinary	[54]
Romania	M2	Codul Deontologic al Farmacistului (Code of Deontology of the Pharmacist, 2009)	Colegiul Farmaciștilor din România (College of Pharmacists of Romania)	Binding + disciplinary	[32]
Slovakia	M1	Kódex etiky zdravotníckych pracovníkov (Code of Ethics for Healthcare Workers), Annex No. 4 to Law No. 578/2004	Legislator (Národná rada/National Council)	Binding + disciplinary	[55]
Slovenia	M2	Kodeks farmacevtske etike (Code of Pharmaceutical Ethics)	Lekarniška zbornica Slovenije (Slovenian Chamber of Pharmacies)	Binding + disciplinary, statutory	[56]
Spain	M2	Código de Deontología de la Profesión Farmacéutica (Code of Deontology of the Pharmaceutical Profession, 2018)	Consejo General de Colegios Oficiales de Farmacéuticos de España	Binding + disciplinary	[23]
Sweden	M3	Lagen om handel med läkemedel (LML, Pharmacy Act, 2009:366) + professional standards	State (Läkemedelsverket/MPA) + Sveriges Farmaceuter-professional association	Voluntary; guidance complementary to statutory regulation	[57,58]
Switzerland	M3	Code de déontologie de la Société Suisse des Pharmaciens (Code of Deontology of the Swiss Pharmacists’ Society)	pharmaSuisse (Société Suisse des Pharmaciens)	Voluntary; complementary to cantonal statutory regulation	[59]
United Kingdom	M1	Standards for Pharmacy Professionals	General Pharmaceutical Council (GPhC)	Binding outcome-based standards	[25]

**Table 3:** Typology of regulatory models for professional error management, continuity of pharmaceutical care, and remuneration of pharmaceutical services across the jurisdictions analyzed.

Area of Regulation	Regulatory Model	Defining Characteristic
Management of professional error	Compensation- and learning-focused	Incident reporting is central; compensation is routed through patient insurance without requiring proof of individual guilt (e.g., Finland).
	Strict personal accountability	The pharmacist remains personally responsible for dispensing and therapeutic safety; malpractice carries direct civil, administrative, or penal consequences (e.g., Estonia, Czechia).
	Institutionally distributed	Error response is shared across statutory rules, chamber ethics, and state oversight rather than a single disciplinary path (e.g., Slovenia).
Continuity of pharmaceutical care	Mandatory public service	Continuity is treated as a non-negotiable state obligation, with state-set duty schedules and financial redistribution or subsidies covering operational costs (e.g., Denmark, Germany).
	Market-adjusted/volunteer	Continuity is assigned to the profession itself, relying on local rotations, collaborative rural obligations, or independent emergency dispensing rights (e.g., Austria, Ireland).
Remuneration of pharmaceutical services	Product-oriented	Income is tied to the margin of the medicine; clinical activities such as counseling are bundled into the dispensing act without separate remuneration (e.g., Estonia, Greece).
	Service-oriented	Professional fees are decoupled from the price of the medicine; pharmacists are remunerated for defined clinical interventions such as medication reviews (e.g., Germany, Belgium).

## Data Availability

The original contributions presented in this study are included in the article. Further inquiries can be directed to the corresponding authors.

## Supplementary Materials

The following supporting information can be downloaded at: https://www.mdpi.com/article/10.3390/healthcare14183028/s1, Table S1: Evidence matrix of 30 European jurisdictions across 14 deontological criteria, with the source of each extracted item and a per-jurisdiction list of consulted instruments. Table S2: SRQR (Standards for Reporting Qualitative Research) compliance map. Reference [159] was cited in the Supplementary Material.

## Abbreviations

The following abbreviations are used in this manuscript:

AI	Artificial Intelligence
AIFA	Agenzia Italiana Del Farmaco
APTA	Employers Association Of Finnish Pharmacies
BPU	Bulgarian Pharmaceutical Union
CJEU	Court Of Justice Of The European Union
COPD	Chronic Obstructive Pulmonary Disease
CPE	Continuing Pharmaceutical Education
DVG	Digitale-Versorgung-Gesetz
EFC	Educatie Farmaceutica Continua
EU	European Union
FIMEA	Finnish Medicines Agency
FIP	International Pharmaceutical Federation
FOFI	Federazione Ordini Farmacisti Italiani
GPHC	General Pharmaceutical Council
GPP	Guidelines On Good Pharmacy Practice
HSE	Health Service Executive
HWG	Heilmittelwerbegesetz
IGJ	Inspectie Gezondheidszorg En Jeugd
INAMI	Institut National D’assurance Maladie-Invalidité
INFARMED	Autoridade Nacional Do Medicamento E Produtos De Saúde
Kela	Kansaneläkelaitos
KNMP	Koninklijke Nederlandse Maatschappij Ter Bevordering Der Pharmacie
LML	Lagen Om Handel Med Läkemedel
MHRA	Medicines And Healthcare Products Regulatory Agency
MPA	Medical Products Agency
NHIFA	National Health Insurance Fund Administration
NICE	National Institute For Health And Care Excellence
OTC	Over-The-Counter
pDL	Pharmazeutische Dienstleistungen
PGEU	Pharmaceutical Group Of The European Union
PSI	Pharmaceutical Society Of Ireland
T2DM	Type 2 Diabetes Mellitus
TEU	Treaty On European Union
TFEU	Treaty On The Functioning Of The European Union
UK	United Kingdom
WHO	World Health Organization
