# Supplementary material for: Professional Independence as the Foundation of Modern Pharmacy Practice: A Comparative Analysis of Deontological Frameworks Across 30 European Jurisdictions
Source: Healthcare (Basel). 2026 Sep 15;14(18):3028. doi: 10.3390/healthcare14183028 (PMC13606226; doi:10.3390/healthcare14183028)
Supplement: Supplementary file 1 [file healthcare-14-03028-s001.zip › Supplementary Table S2.pdf]

Supplementary Table S2 – Standards for Reporting Qualitative Research (SRQR)

Compliance map

No	Topic	Where addressed
S1	Title	Title—identifies the study as a comparative analysis of deontological frameworks across 30 jurisdictions
S2	Abstract	Abstract
S3	Problem formulation	Section 1
S4	Purpose or research question	Section 1, closing paragraph
S5	Qualitative approach and research paradigm	Section 2—qualitative document analysis with comparative legal design; the guiding theoretical framework is presented in Section 1
S6	Researcher characteristics and reflexivity	Section 2 – authors' role in instrument development, extraction and verification procedures, qualification of the author who reviewed the classification
S7	Context	Sections 1 and 2: EU plus UK, Norway and Switzerland
S8	Sampling strategy	Section 2 – documents were included if issued by a national authority or a professional body and if they address one or more of the 14 criteria. No sampling has been applied. All 30 jurisdictions were covered.
S9	Ethical issues pertaining to human subjects	Not applicable—the study analyses publicly available normative documents; no human participants
S10	Data collection methods	Section 2 – extraction January-February 2026; analysis and verification April-June 2026 and August 2026
S11	Data collection instruments and technologies	Section 2 and Table 1 – 14-criterion fixed template; Google Translate, DeepL, Reverso
S12	Units of study	Section 2 and Supplementary Table S1–30 jurisdictions/14 criteria
S13	Data processing	Section 2—translation, source-level and content-level verification of data integrity
S14	Data analysis	Section 2 and Section 3.1 – inductive development of the 5 thematic clusters and 3 model classification
S15	Techniques to enhance trustworthiness	Section 2 – two-stage verification of all 30 jurisdictions, review of the classification by an author with a doctorate in law, audit trail (Supplementary Table S1)
S16	Synthesis and interpretation	Sections 3 and 4
S17	Links to empirical data	Section 3 and Supplementary Table S1
S18	Integration with prior work, implications, transferability, contribution	Sections 4.1-4.5
S19	Limitations	Section 4.6
S20	Conflicts of interest	Conflict of interest statement
S21	Funding	Funding statement

Source for the compliance map template:

Bridget C O'Brien, Ilene B Harris, Thomas J Beckman, Darcy A Reed, David A Cook, Standards for Reporting Qualitative Research: A Synthesis of Recommendations, *Academic Medicine*, Volume 89, Issue 9, September 2014, Pages 1245–1251, https://doi.org/10.1097/ACM.0000000000000388
